# Preparation of Time-Sequential Functionalized ZnS-ZnO Film for Modulation of Interfacial Behavior of Metals in Biological Service Environments

**DOI:** 10.3390/biom14081041

**Published:** 2024-08-22

**Authors:** Jianwen Zhang, Yujie Tang, Xiaowa Gao, Xinyu Pei, Yajun Weng, Junying Chen

**Affiliations:** 1 Key Laboratory of Advanced Technology of Materials, Ministry of Education, School of Materials Science and Engineering, Southwest Jiaotong University, Chengdu 610031, China; zjw.swjtu.edu.cn@my.swjtu.edu.cn (J.Z.); tangyj200@my.swjtu.edu.cn (Y.T.); gaoxiaowa@my.swjtu.ecu.cn (X.G.); xinyupei@my.swjtu.edu.cn (X.P.); 2 Institute of Biomedical Engineering, College of Medicine, Southwest Jiaotong University, Chengdu 610031, China; wengyj7032@swjtu.edu.cn

**Keywords:** ZnS-ZnO film, time-sequential functionalized, superhydrophobic, H_2_S gas, anticoagulation, low adhesion

## Abstract

Blood-contact devices are prone to inflammation, endothelial dysfunction, coagulation, and the uncontrolled release of metal ions during implantation and service. Therefore, it is essential to make these multifunctional. Herein, a superhydrophobic DE@ZnS-ZnO@SA film (composed of dabigatran ester, zinc sulfite, zinc oxide, and stearic acid, respectively) is produced. The prepared film has non-adhesion and antibacterial properties, superior mechanical stability, durability, corrosion resistance, and is self-cleaning and blood-repellent. The results of the hemolysis, cytotoxicity, and other anticoagulant experiments revealed that the film had good blood compatibility, no cytotoxicity, and excellent anticoagulant properties. The film displays anticoagulant properties even after being immersed in Phosphate-Buffered Saline (PBS) for 7 days. Furthermore, the film can spontaneously release H_2_S gas for 90 h after soaking in an acidic environment (pH = 6) for 90 h. This property improves the acidic microenvironment of the lesion and promotes the proliferation of endothelial cells by using H_2_S gas. In addition, the film can inhibit the uncontrollable release of Zn^2+^ ions, avoiding its toxicity even when immersed in an acid environment for 35 days. This time-sequential functionalized surface has the potential to typify the future of blood-contacting scaffolds for long-lasting use.

## 1. Introduction

With increased demand for blood-contact medical devices, such as implantable interventional catheters, vascular stents, and extracorporeal circulation equipment, it is desirable to endow medical devices with multiple functionalities to achieve better biocompatibility, and long-term anticoagulant, antibacterial, and antifouling properties. However, no such multifunctional medical device has been developed yet that could be used in the blood environment for a long time without causing adverse reactions. Most devices have limitations in practical applications, such as the inability to interact with the implanted environment, the lack of environmental adaptability, and single functionality [1]. Further, hemolysis, infection, thrombosis, and thrombosis-related complications are the other common problems of using devices for clinical applications [2,3,4]. When the device comes into contact with the blood, the plasma protein quickly adsorbs on its surface, forming a protein adsorption layer that induces platelet activation. This process usually leads to plasma dysfunction, inflammatory response, and thrombosis [5]. The devices also release metal ions in the blood due to the device surface’s poor mechanical stability and corrosion resistance, leading to inflammatory events, which result in delayed endothelialization and further coagulation [6,7,8]. Various strategies have been considered to alleviate the drawbacks of using these devices. A strategy to improve the anticoagulant activity involved modifying the surface of the instrument by using bioinert polymers, anticoagulants (heparin), catalysts (the catalyzed release of NO), or changing the surface charge, chemical affinity, and hydrophilicity [9]. Typically, anticoagulant heparin is the primary choice for clinical treatment. However, due to the depletion and degradation of heparin molecules at the device’s contact site, the anticoagulant activity is lost, leading to poor durability. Moreover, device users need to take long-term or lifelong anticoagulants, some of which have been shown to cause problems such as thrombocytopenia or bleeding [10]. In addition, various fungicides and antibiotics are widely used in clinical practice to combat infection and have been shown to reduce the risk of infection to some extent. However, the long-term use of fungicides and antibiotics may lead to abnormal bleeding, bacterial resistance, toxicity, liver burden, and other side effects [11]. Notably, local administration to the lesion site reduces the drug’s toxicity and side effects on the body, and provides more lasting anticoagulant and antibacterial properties than systemic administration.

Vascular stents in blood-contacting devices, as a typical representative of local administration, damages the endothelial function when implanted at the lesion site, deepening the endothelial dysfunction at the lesion site, which leads to thrombosis and new atherosclerosis [12,13]. Therefore, an ideal vascular stent should be able to promote rapid endothelialization and prevent coagulation. Hydrogen sulfide (H_2_S), as a vascular protective gas transmitter, can promote angiogenesis by promoting the proliferation and migration of endothelial cells (ECs) [14,15]. However, the delivery rate and dose of gaseous H_2_S need to be strictly controlled, since the short half-life of H_2_S limits the therapeutic delivery of deep lesions. Therefore, the precise regulation of H_2_S delivery at the pathological site is the key to maximizing the therapeutic effect. Sodium sulfide (Na_2_S) and sodium hydrogen sulfide (NaHS) are the most common inorganic H_2_S-releasing compounds. They can rapidly increase the concentration of H_2_S. However, they release H_2_S as soon as they come into contact with an aqueous solution, making it difficult to simulate endogenous H_2_S and to control its concentration. Consequently, the concentration of H_2_S in solution rapidly fluctuates as it is released spontaneously and volatilizes [16]. Thus, insoluble sulfides such as ZnS were selected as H_2_S donors to overcome this limitation. They exhibit good biocompatibility and can be used as a corrosion inhibitor to improve the corrosion resistance of the scaffold and respond to the acidic pathological microenvironment to release H_2_S, thereby improving the pathological microenvironment and promoting endothelial cell growth [17,18]. Therefore, it is the need of the hour to develop a vascular scaffold with good mechanical stability, corrosion resistance, and biocompatibility to achieve long-term anticoagulation, antibacterial properties, and endothelialization.

Recently, superhydrophobic surfaces have been considered ideal blood-compatible biomaterials due to their excellent properties, such as self-cleaning, corrosion resistance, being antibacterial, and drug delivery [19]. These properties are attributed to the fact that the extremely low surface energy and rough micro/nanostructures of the superhydrophobic surface reduce the effective surface area that can be combined between the platelets, red blood cells, proteins, and solids in the blood. They also reduce the shear stress on the surface. Consequently, the adhesion between the blood components is significantly reduced, reducing the damage caused by the interaction of blood cells on the instrument’s surface [20,21]. These characteristics have overcome most medical devices’ long-standing blood compatibility problems. Additionally, the antibiofouling effect of superhydrophobic surfaces is also expected to be used in medical devices because it can resist bacterial attachment, reducing their viability [22,23]. This is due to the air layer intercepted by the superhydrophobic surface at the solid–liquid interface, which acts as a barrier between the substrate and the liquid below, significantly reducing microbial adhesion [24]. Moreover, reducing the surface energy through surface modification also helps with self-cleaning, which is conducive to the detachment and removal of bacterial cells.

The superhydrophobic surface can also improve the instrument’s mechanical stability, corrosion resistance, and durability in a complex blood environment, effectively preventing the shedding of harmful substances and the release of metal ions [25,26,27]. However, the current research on superhydrophobicity is limited to a single functional regulation. There are few reports on multifunctional blood-contact devices with antifouling, anticoagulant, antibacterial, endothelialization, corrosion resistance, and mechanical stability. Fluorine-containing materials are widely used in preparing superhydrophobic surfaces due to their extremely low surface energy. However, fluoride is expensive, has poor economic benefits, and is reported to cause cancer and other health hazards. These defects seriously hinder the applications of superhydrophobicity in clinical treatment [28,29]. Therefore, finding a low-surface-energy compound with high blood compatibility, non-biological toxicity, and stability is necessary to enable superhydrophobic blood-contact instruments to promote endothelialization, anticoagulation, antibacterial, and durability. Stearic acid (SA) and dabigatran ester (DE) are ideal candidates for the creation of superhydrophobic surfaces due to their inherent hydrophobicity and biocompatibility, as demonstrated in [30]. Furthermore, DE’s anticoagulant properties, as detailed in [31], make it a particularly valuable component for applications in the medical field.

In this study, a ZnS-ZnO film with a multilayer dense microsphere structure was grown in situ on the Zn substrate surface by a simple one-step hydrothermal method. Further, the surface was modified by SA and DE to prepare a multifunctional superhydrophobic film. The resultant DE@ZnS-ZnO@SA film has excellent superhydrophobicity, low adhesion, is self-cleaning, and has mechanical durability and corrosion resistance. The results of the in vitro biological toxicity experiments demonstrated that the film had good biocompatibility and no cytotoxicity. The film was also found to have excellent anticoagulant properties through dynamic coagulation, platelet adhesion, activation, in vitro whole-blood static culture, and half-body dynamic circulation experiments. The film exhibited good anticoagulant properties even after being immersed in Phosphate-Buffered Saline (PBS) for 7 days. Further, the film showed good inhibition and antibacterial activity due to its low adhesion and the release of Zn^2+^. Most importantly, the slow and controlled in situ release of H_2_S in an acidic microenvironment (pH = 6) provides a continuous pro-endothelialization effect. In summary, when the film is used in the acidic lesion microenvironment, the blood-repellent behavior of the superhydrophobic surface and the release of the anticoagulant DE make the film behave like a long-term anticoagulant.

## 2. Experimental Section

### 2.1. Materials

Zinc nitrate hexahydrate (Zn(NO_3_)_2_·6H_2_O, 99.99%, CSA:10196-18-6), thiourea (CH_4_N_2_S, 99%, CSA:62-56-6), dabigatran etexilate (DE, C_34_H_41_N_7_O_5_, 97%, CSA:211915-06-9), and stearic acid (SA, C_18_H_36_O_2_, 99%, CSA: 57-11-4) were purchased from Macklin, Shanghai, China. These chemicals were used as-is without further purification.

### 2.2. Fabrication of Superhydrophobic ZnS-ZnO@SA and DE@ZnS-ZnO@SA Composite Films

The detailed process of preparing the ZnS-ZnO composite film by the hydrothermal method has been reported in our previous work [3,17]. The specific steps are as follows. The Zn substrate (99.99% pure) was cropped into 20 mm × 25 mm × 0.25 mm and ultrasonically cleaned in detergent solution and absolute alcohol for 30 min each to remove surface grease. Chemical polishing was performed in 0.75 M NaOH solution for 40 min to eliminate surface defects, resulting in a smooth surface. Next, zinc nitrate hexahydrate (0.3 g) and thiourea (0.24 g) were added to 40 mL of deionized water with ultrasonic stirring until a transparent precursor solution was obtained. Next, the polished Zn substrate was placed in a Teflon-lined stainless-steel autoclave filled with 30 mL of precursor solution to initiate the hydrothermal synthesis. The reaction temperature was adjusted from 100 °C to 180 °C, and the corresponding reaction time was controlled from 4 h to 16 h. After the hydrothermal reaction, the sample was removed, washed with deionized water, and dried using a blower to obtain the ZnS-ZnO film. Finally, the ZnS-ZnO film was soaked in 7 mmol/L SA solution for 30 s to coat the SA on the surface of the ZnS-ZnO film. Then, the superhydrophobic ZnS-ZnO@SA composite films were obtained.

In order to further enhance the function of the ZnS-ZnO@SA film, anticoagulant DE and SA were co-modified on the surface of the ZnS-ZnO film. The experimental steps were as follows: 16 mg of DE and 0.05 g of stearic acid were dissolved in 20 mL of ethanol by ultrasonic stirring. Finally, the ZnS-ZnO composite film was immersed in a mixed solution containing stearic acid (SA) and dabigatran ester (DE), and placed in a thermostatic shaker for 24 h (37 °C, rotation speed 80 r/min). The superhydrophobic DE@ZnS-ZnO@SA film was obtained after drying at 50 °C for 4 h.

### 2.3. Characterization

The chemical composition and crystal structure were analyzed using Fourier transform infrared spectroscopy (FTIR, Semirfei, Nicolet iS50, San Diego, CA, USA) and X-ray diffraction (XRD, Empyrean, Malvinpanaco, Shanghai, China). The surface morphologies of the synthesized samples were observed via scanning electron microscope (SEM, JSM 7800F Prime, Japan Electronics, Tokyo, Japan). Energy-dispersive spectroscopy (EDS, Energy Spectrum OXFROD X-Max 80, Japan Electronics) was used for determining the surface element compositions. The surface chemical state was examined using X-ray photoelectron spectroscopy (XPS, Thermo Scientific ESCALAB Xi+, Waltham, MA, USA). The cumulative release of Zn^2+^ was measured using an atomic absorption spectrophotometer (TAS-990F, Beijing General Analysis, Beijing, China). The photoluminescence (PL) spectra were tested by the fluorescence spectrophotometer (Hitachi-F4600, Tokyo, Japan). The water contact angle (WCA) and adhesion were determined by averaging multiple measurements in random positions at room temperature using the LSA100 system with a 5 μL water droplet (LAUDA Scientific, Lauda-Königshofen, Germany). The surface energy was calculated by sending the sample to a special test facility (e ceshi(www.eceshi.com (accessed on 18 July 2024))). The corrosion resistance was evaluated by measuring the potentiodynamic polarization (Chenhua CHI E660, Shanghai, China) in 3.5 wt% NaCl aqueous solution through an electrochemical workstation. All optical videos and photos were obtained using a digital camera.

### 2.4. Hydrogen Sulfide Release Profile at Different pH Values

The in vitro H_2_S gas release profile of the ZnS-ZnO@SA film was investigated at different pH values to analyze the H_2_S release properties. The ZnS-ZnO film was immersed in 10 mL of PBS at various pH values (pH = 5, 6, and 7). At each incubation interval (up to 24 h), 500 μL of the sample solution was collected and replaced with fresh PBS at the required pH level. The collected H_2_S-containing solution was mixed with a zinc acetate/sodium acetate mixture (4:1 mass ratio, 1 mL). Methylene blue (MB) was then prepared by adding N, N-dimethyl-p-phenylenediamine dihydrochloride dye (2.5 mM) and FeCl_3_ (3.5 mM), respectively. After incubation for 15–20 min, the absorbance at 670 nm was measured, and the concentration of H_2_S was determined using a standard curve of the Na_2_S solution.

### 2.5. Anti-Blood Adhesion Test

All animal procedures complied with the Southwest Jiaotong University Guide for the Care and Use of Laboratory Animals and were approved by the Animal Ethics Committee of the Southwest Jiaotong University Ethics Committee (SWJTU-2203-NSFC (003)). Fresh whole blood was obtained from a New Zealand White rabbit. During the dynamic blood clotting test, fresh whole blood (100 μL) without anticoagulant was dropped on the sample surface (10 mm × 10 mm) and placed in the wells of a 24-well cell culture plate. After incubation for 10, 20, 30, and 40 min at 37 °C, 1 mL of deionized water was added and soaked for 5 min. Finally, the absorbance (OD) of the blending solution was determined at 540 nm using a microplate reader (Varioska LUX, Thermo, Waltham, MA, USA). The adhesion of the whole blood and platelets on the sample surface was observed using the SEM. First, the fresh whole blood was centrifuged at 1500 rpm for 15 min to obtain platelet-rich plasma. The samples (10 mm × 10 mm) were incubated with whole blood (500 μL) and platelet-rich plasma (500 μL) at 37 °C for 45 min, respectively. Then, the non-adherent whole blood and platelets were removed with 0.9 wt% NaCl solution. Subsequently, the sample was fixed with glutaraldehyde, and the SEM characterization was performed after the dehydration treatment. The blood repellency test was performed by dripping the fresh blood onto the film surface using a dropper several times.

### 2.6. Antibacterial Property

The antibacterial activity of the Zn@SA, ZnS-ZnO, ZnS-ZnO@SA, and DE@ZnO-ZnS@SA films’ surfaces was enforced via the inhibition ring method. *S. epidermidis* and *E. coli* were selected as the type bacterial strains. The Zn@SA, ZnS-ZnO, ZnS-ZnO@SA, and DE@ZnO-ZnS@SA composite films were cut into a square (10 mm × 10 mm), followed by UV irradiation for 30 min for sterilization. The bacterial suspension (30 μL, 10^6^ CFU/mL) was consistently spread on an LB agar surface, and then the square samples were placed onto them. After 24 h of incubation at 37 °C, the zone of inhibition was measured. Also, the antibacterial property was further explored via agar plate colony counting. The sample (10 mm × 10 mm) was immersed in bacterial suspension (10^5^ CFU/mL) and incubated at 37 °C for 24 h. After 24 h of culturing, 50 μL of the bacterial solution in each sample was coated on the surface of a solid medium to count the number of colonies. The antibacterial rate was calculated by the following formula:

R (%) = (1 − (N_e_/N_c_)) × 100

where R is the antibacterial rate, N_c_ is the number of colonies in the control group, and N_e_ is the number of colonies in the experimental group.

### 2.7. Cell Compatibility Evaluation

The cytotoxicity of the Zn, ZnS-ZnO, ZnS-ZnO@SA, and DE@ZnS-ZnO@SA films was evaluated via the CCK-8 cell viability assay. Briefly, the samples were thoroughly disinfected and placed in a 24-well culture plate; then, the cells in the medium were seeded on the surface of each sample with 2 × 10^4^ to 3 × 10^4^ cells per well, and cultured at 37 °C and in a 5% CO_2_ incubator for 24 and 72 h. Cell viability was analyzed via calcein staining and the cell counting KIT-8 (CCK-8). The CCK-8 kit was used to detect the cell activity. The CCK-8 reagent was mixed with the cell culture medium at 1:9, and 300 μL was added to the orifice plate containing only the sample and ECs. After incubation in the incubator at 37 °C for 2 h, the absorbance value was detected at 450 nm with a microplate reader.

### 2.8. Statistical Analysis

Data for the WCA, in vitro dynamic blood clotting time, and inhibition zone analysis data were expressed as the mean ± standard deviation (SD) of the three samples. All of the measurement results were expressed as the mean ± standard deviation (SD) at least in triplicate. All of the statistics were analyzed using Origin 8.5. When the *p* value was less than 0.05, the difference between the sample groups was considered to be significant; *, **, and *** denote *p* < 0.05, *p* < 0.01, and *p* < 0.001, respectively.

## 3. Results and Discussion

### 3.1. Morphology, Physical Properties, and Composition Characterization

**Figure 1:**
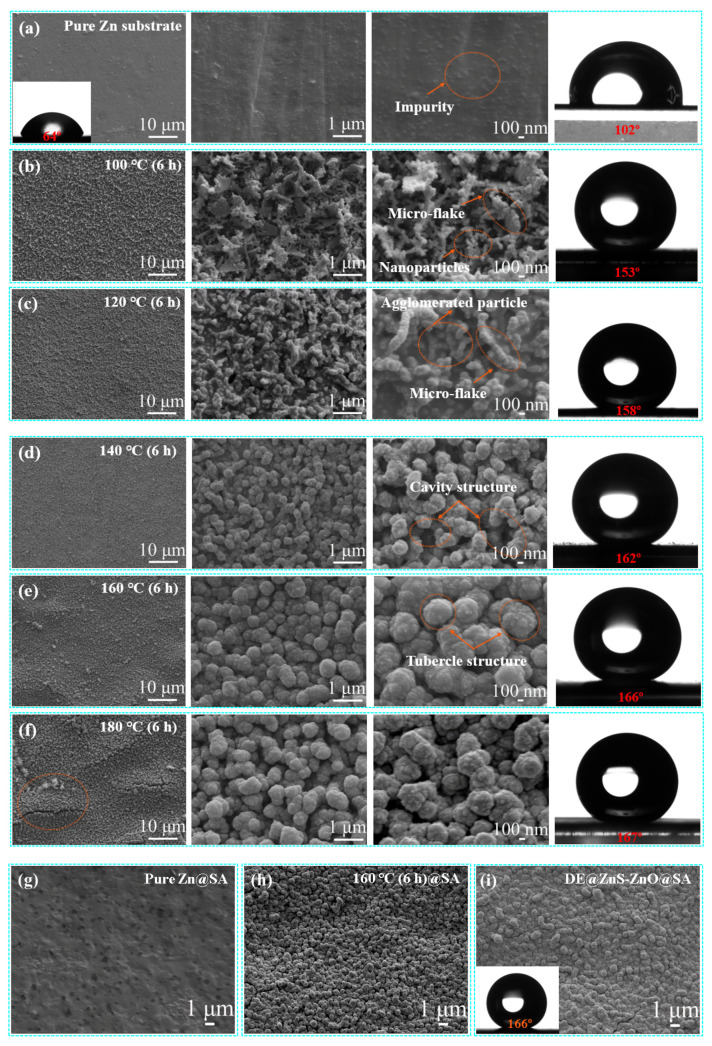
(**a**) The SEM images of the Zn substrate and WCA image after the SA modification. The SEM images of the hydrothermal synthesized sample surface under the different hydrothermal temperatures of (**b**) 100 °C, (**c**) 120 °C, (**d**) 140 °C, (**e**) 160 °C, and (**f**) 180 °C, a hydrothermal time of 6 h, and the WCA image after the SA modification on the corresponding surface. (**g**,**h**) The SEM images of the pure Zn substrate and ZnS-ZnO (160 °C) surface with the SA modification. (**i**) The SEM image of the ZnS-ZnO surface with the SA and DE modification (the concentration of DE was 800 μg/mL).

The morphology of the samples at different magnifications was observed by the SEM to understand the effect of the hydrothermal time and temperature on the micro/nanostructures and surface wettability. As shown in Figure S1a–d, the constant hydrothermal reaction temperature was controlled at 160 °C, and the ZnS-ZnO film showed slightly variable morphologies with an increased hydrothermal time from 4 h to 16 h. The longer the hydrothermal time (16 h), the higher the number of cracks and discrete petals that appeared in the film, showing a clear hierarchical structure; however, the film structure was not dense, uniform, and regular. There was no significant difference in the wettability of the film surface after the SA modification, and the water contact angle (WCA) was greater than 161° (Figure S1e–h). Figure 1a shows the SEM image of the Zn substrate surface after polishing with sodium hydroxide. The polished Zn substrate shows a relatively smooth surface, as indicated by a WCA of 64°. The WCA after the SA modification was 102°. When the hydrothermal temperature was 100 °C, a film with a rough structure was formed on the surface of the Zn substrate. The magnified image shows that the film was composed of nanoparticles and irregular cross-micro-sheets formed by the accumulation of nanoparticles. The particle size was about 80 nm (Figure 1b). The film showed an apparent layered structure, and the formed pits and protrusions caused a significant gap in the film. The WCA reached 153° after the SA modification. As the hydrothermal temperature increased to 120 °C, the nanoparticles at the pit position grew preferentially, the particle size gradually increased, and agglomeration occurred, making the film denser without apparent voids. The particle size was about 120 nm. The original cross-micro-sheets were covered by newly generated nanoparticles at the bottom of the pit, forming a relatively uniform granular film. The WCA after the SA modification was 158° (Figure 1c). As the hydrothermal temperature increased to 140 °C, the nanoparticles continued to grow, and the arrangement of secondary nanoparticles on the primary nanoparticles was observed to be more uniform and regular. The particle size was about 220 nm. The particles formed an irregular submicron particle film in the form of a stacked mohan. The WCA after the SA modification was 162° (Figure 1d). When the hydrothermal temperature increased to 160 °C, all of the submicron spheres were aggregated to form a uniform and dense micron-scale mastoid structure, and the surface morphology became more regular and orderly, masking the cavity structure formed in the irregular submicron particle film (Figure 1e). The particle size was about 410 nm. The mastoid structure made the film appear concave and convex in texture, similar to the morphology of the lotus leaf surface, and the WCA reached 166° after the SA modification. However, when the hydrothermal temperature was further increased to 180 °C, the overall morphology and structure did not change much (compare Figure 1f with Figure 1e); however, there were apparent microcracks on the surface of the film, and there were some micron petals near the cracks (As shown in the red circle mark). The particle size was about 390 nm. The WCA reached 167° after modification with the SA. Thus, 180 °C was the critical temperature for the growth of petal-like structures. Also, the cross-section SEM image and enlarged image of the sample with a hydrothermal temperature of 160 °C showed that the ZnS-ZnO film was closely integrated with the substrate. It was composed of multilayer nano–submicron–micron particles (Figure S1i). Therefore, the nano–submicron–micron rough structure regulation was realized by controlling the hydrothermal temperature, and the film exhibited some noticeable wettability differences. The wettability results showed that the WCA gradually increased with the temperature. Based on this analysis and comparison, the ZnS-ZnO film with a hydrothermal temperature of 160 °C and hydrothermal time of 6 h was used for the subsequent experiments and analyses.

Also, compared with the micro-morphology of the SA-modified Zn substrate and ZnS-ZnO film (Figure 1g,h), the Zn substrate and ZnS-ZnO film modified by the SA did not change their morphologies, and they still had flat surfaces and microsphere films. The voids between the microspheres filled with air, which was conducive to forming air cushions and contributed to the subsequent research of the superhydrophobic film. However, the wettability of the sample was significantly related to whether it was modified with the SA. The WCA of the untreated Zn substrate was 64°, which was hydrophilic. The WCA of the ZnS-ZnO film prepared at different hydrothermal temperatures without the SA modification was less than 10°, indicating superhydrophilicity (Table S1). The effect of loading anticoagulant on the original morphology of the ZnS-ZnO film was further investigated. The combination of DE and SA did not change the original rough structure, and the WCA reached 166° (Figure 1i). The high-power scanning electron microscope showed that only a thin layer of wax-like material was wrapped on the surface of the mastoid structure after loading the anticoagulant (Figure S1j).

**Figure 2:**
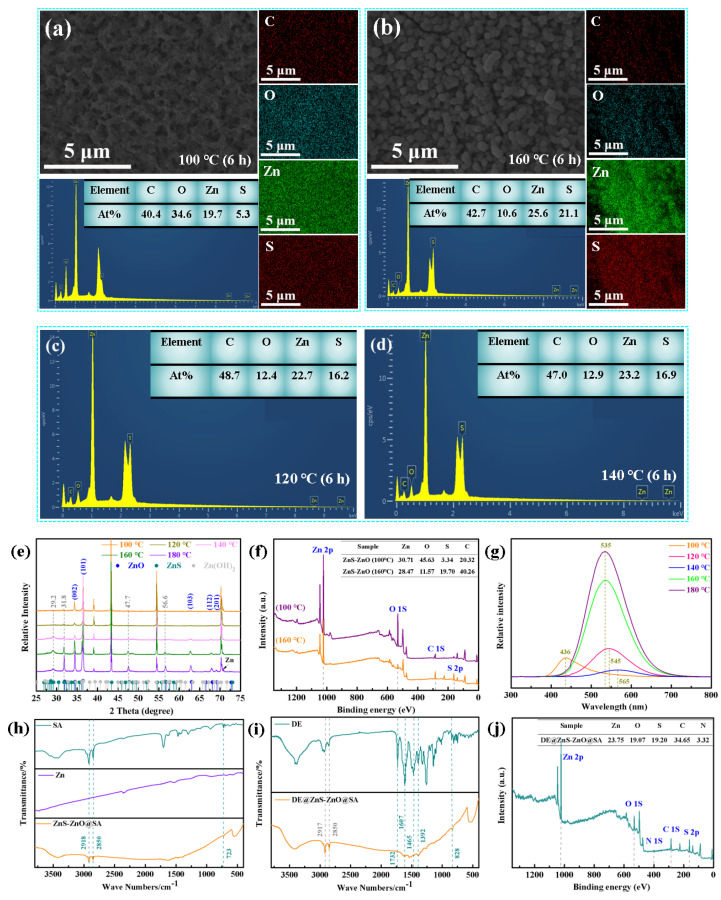
(**a**,**b**) The SEM images, EDS, and surface element mappings of the ZnS-ZnO film under the hydrothermal temperatures of 100 °C and 160 °C. (**c**,**d**) The EDS of the film prepared at hydrothermal temperatures of 120 °C and 140 °C. (**e**,**g**) The XRD patterns and PL spectra of the ZnS-ZnO film surface with different hydrothermal temperatures. (**f**,**j**) The XPS survey spectra of the ZnS-ZnO film with hydrothermal temperatures of 100 °C and 160 °C, and the DE@ZnS-ZnO@SA surface. (**h**,**i**) The FTIR spectra of the pure SA, DE, Zn, ZnS-ZnO@SA, and DE@ZnS-ZnO@SA surfaces.

Further analysis of the energy-dispersive spectrum (EDS) of the ZnS-ZnO film at different hydrothermal temperatures showed that the hydrothermal temperature affected the content of ZnS and ZnO on the film surface. There were carbon, oxygen, zinc, and sulfur elements in the surface structure at different hydrothermal temperatures (Figure 2a,b), and the uniform distribution of the element mappings confirmed that the film was composed of ZnS and ZnO; however, the main content of elements on the film was very different. When the hydrothermal temperature was 100 °C, the element content on the film’s surface showed that the O element was significantly higher than that of the S element, proving that ZnO was the primary constituent of the film. When the hydrothermal temperature was 160 °C, the content of the S element on the surface of the film was significantly higher than that of the O element, indicating that, with the increase in the hydrothermal temperature, the content of ZnS gradually increased, and the primary constituent on the surface of the film became ZnS. In order to further confirm this result, EDS tests were performed on the samples with hydrothermal temperatures of 120 °C and 140 °C. These results showed that the content of the S element in the sample was higher than that of the 100 °C hydrothermal treatment sample, but lower than that of the 160 °C sample. Therefore, with the increase in the hydrothermal temperature, the primary constituent of the film surface changed from ZnO to ZnS, forming a composite structure composed of ZnS and ZnO.

Based on the results of the EDS, the crystal phase’s evolution on the film’s surface was further investigated by measuring the XRD patterns of the samples synthesized at different hydrothermal temperatures. Compared with the XRD standard cards of ZnS and ZnO (JCPDS Card NO. 39-1369 (sphalerite structure of ZnS) and JCPDS Card NO. 36-1451 (Wurtzite structure of ZnO)), the prominent characteristic peaks and relative intensities of the ZnS and ZnO were consistent with the XRD standard cards, indicating that the film was composed of ZnS and ZnO composite films (Figure 2e). Additionally, weak mixed peaks of Zn(OH)_2_ and a small amount of metal Zn impurity peaks also simultaneously appeared in the XRD patterns. The appearance of the metal Zn impurity peak may be caused by the interference of the Zn substrate during the detection process. In addition, in the experimental section, the ZnO was formed mainly through the chemical reaction of Zn + H_2_O → Zn(OH)_2_ + H_2_ and Zn(OH)_2_ → ZnO + H_2_O. This process will produce an incomplete reaction of Zn (OH)_2_, but its content was very low, which will not affect the main research content of the paper [32]. Also, by performing an in-depth analysis of the XRD results at different hydrothermal temperatures, the intensity of some characteristic peaks (29.2°, 31.8°, 47.7°, and 56.6°) corresponding to the ZnS diffraction peaks gradually increased with an increase in the hydrothermal temperature, further proving that the content of ZnS increased with an increase in the hydrothermal temperature, which was consistent with the results of the EDS. Due to a noticeable difference in the structure and surface composition of the samples at the hydrothermal temperatures of 100 °C and 160 °C, the surface chemical state of the two samples was further analyzed via X-ray photoelectron spectroscopy (XPS) (Figure 2f). The Zn 2p, S 2p, C1 s, and O 1s signal peaks appeared in the XPS measurement spectra of the ZnS-ZnO film at both temperatures, indicating that the ZnS-ZnO film was successfully prepared. The element content showed that the O element content of the 160 °C sample was significantly lower than the O element content of the 100 °C sample; however, the S element content was significantly higher than the S element content in the 100 °C sample, indicating that the increase in the hydrothermal temperature increased the content of ZnS, which was entirely consistent with the results of the EDS and XRD analyses. Also, in the high-resolution XPS spectrum of Zn 2p (Figure S2a), two individual peaks near 1021.7 and 1044.7 eV were observed, which were related to the Zn 2p_3/2_ and Zn 2p_1/2_ spin orbits of Zn^2+^ in ZnO and ZnS, respectively. Compared with the 100 °C sample, the Zn 2p peak of the 160 °C sample shifted to the low binding energy due to the interaction between ZnS and ZnO. The binding energy difference between the Zn 2p_1/2_ and Zn 2p_3/2_ of the two samples was ~23 eV, and the interval of 23 eV signified that the Zn element in the samples was mainly present in Zn^2+^ [33]. The O 1s peak was decomposed into three components with binding energies of 530.8 eV, 531.4 eV, and 532.0 eV, respectively, which were attributed to the formation of the Zn-O bond, adsorbed oxygen in the air, and -OH on the ZnO surface [34] (Figure S2b). Figure S2c shows a high-resolution S 2p spectrum. The 100 °C and 160 °C samples had three fitting peaks at 161.5 eV and 162.7 eV, two of which were attributed to S 2p_3/2_ and S 2p_1/2_. These two fitting peaks were related to the Zn-S bonding. Also, the S 2p spectrum was fitted to a distinct sub-peak at 161.9 eV, representing the chemical state of sulfur in S^2−^ [32]. These characterization results confirm that the crystal phase of ZnS begins to dominate at 120 °C, which was consistent with the results of the XRD and EDS analyses. Also, the PL spectra at room temperature proved that the ZnS-ZnO film was a composite film. The samples with 100, 120, 140, 160, and 180 °C hydrothermal temperatures were measured. The excitation wavelength was 325 nm, and the measurement range was 300–800 nm. The results showed that, with an increase in the hydrothermal temperature, the position and intensity of the luminescence peak increased first and then decreased. On the one hand, this may be due to the difference in the morphology and composition of the samples. On the other hand, this may be due to the energy level matching between the materials and the different electron/hole recombination mechanisms (Figure 2g) [35]. Also, different growth environments and crystal surface environments produced different lattice defects, which were reflected in the defect peaks of the photoluminescence spectra, and shifted the center of the visible light band. In addition, for the composite materials, due to the lattice mismatch of the different materials or the stress at the interface, the width of the luminescence peak may change [36]. Emission-related defects are usually known to occur at blue–green wavelengths (480–550 nm), yellow wavelengths (550–610 nm), and orange wavelengths (610–750 nm) [37]. The photoluminescence properties of the ZnS-ZnO composite film were regulated by changing the morphology and structure of the ZnS-ZnO composite film. The FTIR spectra of the pure SA, pure Zn, and superhydrophobic ZnS-ZnO@SA surfaces were measured to further explore the functional groups on the surface of the superhydrophobic sample (see Figure 2h). Compared with the infrared spectra of the pure SA, the absorption peaks of the ZnS-ZnO@SA surface at 2918 and 2850 cm^−1^ were completely consistent with the characteristic peaks of the SA, but did not appear in the spectra of the superhydrophilic ZnS-ZnO surface, confirming that the surface of the film had been successfully modified by the SA. Also, the infrared spectra of the pure DE were tested to prove that the film was successfully loaded with anticoagulants. The drug-loaded film showed characteristic peaks corresponding to pure DE at 828, 1392, 1465, 1607, and 1732 cm^−1^ (Figure 2i), and distinct peaks of SA at 2917 and 2850 cm^−1^, which proved that the ZnS-ZnO film was successfully loaded with the DE and SA. The XPS further analyzed the elemental composition of the surface of the DE@ZnS-ZnO@SA. The XPS spectra showed that the nitrogen element appeared on the film surface, and the element content was 3.32%, further proving that the film was successfully loaded with anticoagulants (Figure 2j). Similarly, the peaks of amino and benzimidazole groups in the DE were also shown in the high-resolution spectrum of the N element, which further proved the successful loading of the DE (Figure S2d). The EDS of the DE@ZnS-ZnO@SA surface was measured to further study whether the DE was evenly distributed on the film surface. The EDS showed that the characteristic elements on the surface of the drug-loaded film were C, O, Zn, S, and N, and the C, O, and N elements were evenly distributed on the film surface, indicating that the ZnS-ZnO film was successfully covered by the DE and SA (Figure S2e).

In addition, since the surface energy is a key property of the surface wettability, the surface free energy of the Zn@SA, ZnS-ZnO, ZnS-ZnO@SA, and DE@ZnS-ZnO@SA film surfaces was further tested. The surface free energy of the Zn@SA film was 38.3 mJ/m^2^, the surface free energy of the ZnS-ZnO film was 49.2 mJ/m^2^, the surface free energy of the ZnS-ZnO@SA film was 26.5 mJ/m^2^, and the surface free energy of the DE@ZnS-ZnO@SA film was 23.4 mJ/m^2^. The results show that the combined action of the SA and hierarchical micro/nanostructures can significantly reduce the surface free energy of the material surface.

### 3.2. Mechanical Durability of the Superhydrophobic DE@ZnS-ZnO@SA Film Surface

**Figure 3:**
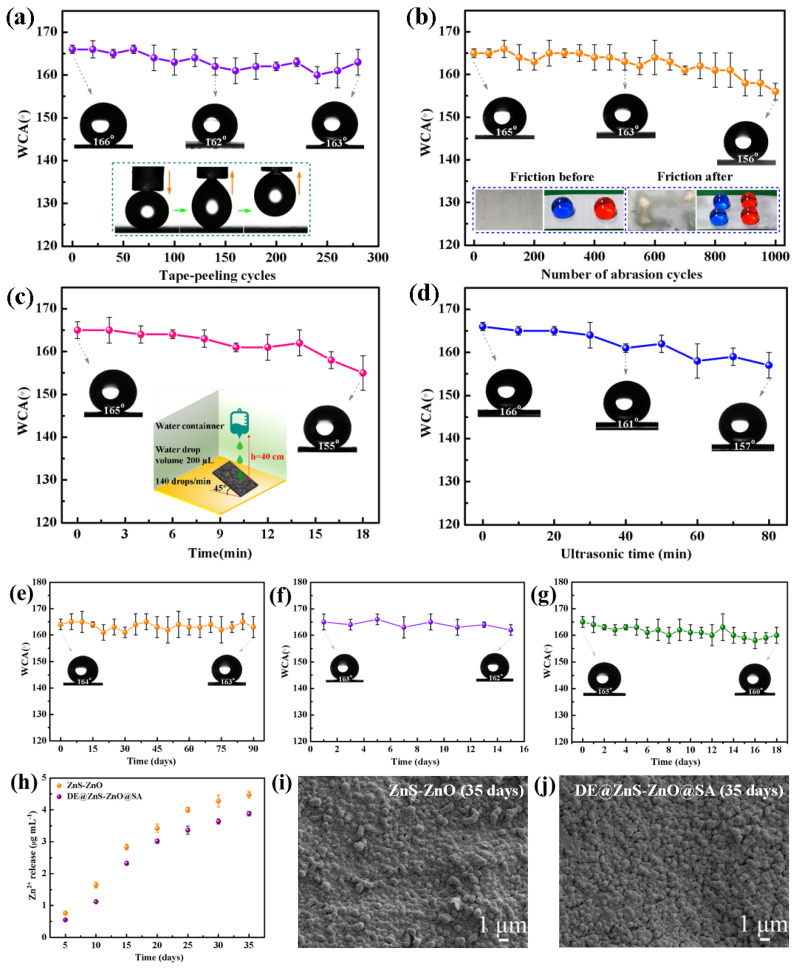
(**a**–**d**) The WCA variation in the DE@ZnS-ZnO@SA film surface with the tape-peeling cycles, abrasion cycles, a water impact test, and ultrasonic treatment time. (**e**,**f**) The WCA variation with exposure time to room temperature storage and UV irradiation for the DE@ZnS-ZnO@SA film surface. (**g**) The WCA variation with the soak time of the DE@ZnS-ZnO@SA film in the water solution. (**h**–**j**) Cumulative release of Zn^2+^ ions and SEM images from the ZnS-ZnO and DE@ZnS-ZnO@SA films after immersion in weakly acidic aqueous solution.

Superhydrophobic surfaces have been widely used to solve blood compatibility disorders such as hemolysis and the platelet activation of implantable or interventional medical devices. High levels of superhydrophobic stability are the main requirement for clinical applications. Therefore, standard tape-peeling, sandpaper abrasion, water impact, and ultrasonic tests were performed on the superhydrophobic DE@ZnS-ZnO@SA surface to verify the mechanical stability of the film. The adhesion strength of the superhydrophobic DE@ZnS-ZnO@SA film to the Zn substrate was evaluated by studying the WCA of the film in the tape abrasion test (Figure 3a). After repeating 280 times, the WCA of the sample still reached 163°, and the water droplets remained spherical on the film’s surface. Also, the illustrations (Figure 3a) show that the film still had a low adhesion force after 280 tape-stripping experiments, indicating that the DE@ZnS-ZnO@SA film closely combined with the Zn substrate and resisted external mechanical damage. Figure 3b shows the change in the WCA on the surface of the DE@ZnS-ZnO@SA film with the number of friction cycles. A 70 g weight was moved horizontally by 5 cm on the surface of 400 mesh sandpaper, and then the sample was moved back to the initial position to complete a friction resistance test cycle. With an increase in the wear time, the static WCA of the film slightly decreased. After the 1000× friction treatment, the WCA of the film decreased to 156°, maintaining its superhydrophobicity, and the water droplets remained spherical on the surface of the film (see the illustration in Figure 3b), indicating that the superhydrophobicity of the DE@ZnS-ZnO@SA film was not significantly damaged under mechanical force. Our prepared DE@ZnS-ZnO@SA film exhibited superior mechanical durability compared to previously reported superhydrophobic surfaces. The latter were observed to transition from a superhydrophobic to a hydrophobic state after a mere 10 cycles of abrasion testing. In contrast, our film maintained its superhydrophobic properties through the same testing regimen, demonstrating a significant enhancement in longevity and robustness for practical applications [38]. This phenomenon occurred because the superhydrophobic DE@ZnS-ZnO@SA film had a hard microsphere convex structure, which could effectively resist external mechanical wear. Also, the superhydrophobic DE@ZnS-ZnO@SA film was subjected to a water droplet impact test (Figure 3c). After impacting the film with a certain amount of water from 40 cm at a speed of 2.85 m/s for 18 min, the water droplets were still spherical on the surface of the film, and the WCA was 155°, indicating that the superhydrophobic DE@ZnS-ZnO@SA film could withstand the impact of the water droplets. This result is comparable to the results of the water shock resistance reported in the literature [39]. These phenomena indicated that the rough structure of the ZnS-ZnO film and the combined action of the hydrophobic materials SA and DE resisted the external impact to a certain extent and maintained its superhydrophobic properties. Also, the superhydrophobic DE@ZnS-ZnO@SA film was placed in an ultrasonic water bath with a power of 100 W to explore the change in the WCA with ultrasonic time (Figure 3d). With an increase in the ultrasonic treatment time, the WCA on the film surface showed a slight downward trend. Even after the ultrasonic treatment for 80 min, the WCA was >157°. Thus, the DE@ZnS-ZnO@SA film had excellent mechanical stability, supporting the long-term stability required for medical devices after implantation into the human body. This is mainly due to the existence of the multilayer nano–submicron–micron particle structure and low-surface-energy SA. This structure protects the SA from wear and gives it a self-healing property in the wear test, ensuring that the surface maintains the original superhydrophobicity and has good mechanical durability. Water impact experiments further proved these results.

In addition, many superhydrophobic films lose their superhydrophobicity after periodic exposure to the air environment. Therefore, it was necessary to test the durability of the DE@ZnS-ZnO@SA film. The sample still showed excellent superhydrophobicity after long-term storage in air for 90 days, and the final WCA remained at 163°, indicating that the prepared superhydrophobic film had exceptional durability and stability (Figure 3e). In addition to durability, the DE@ZnS-ZnO@SA film also showed excellent UV resistance. When the film was placed under UV light at 302 nm for 15 days, the surface WCA almost showed no change and maintained its superhydrophobic properties, proving its good UV resistance (Figure 3f). The DE@ZnS-ZnO@SA film was further immersed in water for 18 days. As shown in Figure 3g, the WCA did not change significantly with the increase in the immersion days, which proved that the film had excellent superhydrophobicity and waterproof ability. The ZnS-ZnO and DE@ZnS-ZnO@SA films were immersed in an acidic aqueous solution for 35 days (pH = 6), and the concentration of Zn^2+^ in the solution at different time points was detected to detect the stability of the DE@ZnS-ZnO@SA film quantitatively. Figure 3h shows the experimental results. The cumulative release of Zn^2+^ from the ZnS-ZnO film (4.48 μg/mL) during the whole immersion process was always higher than the concentration of Zn^2+^ released from the DE@ZnS-ZnO@SA film (3.88 μg/mL), indicating that the superhydrophobicity reduced the degradation rate of Zn^2+^. The cumulative release of Zn^2+^ from our films, when compared to the literature values up to 15 μg/mL, has been shown not to induce toxicity in cells. This finding substantiates the excellent cell compatibility of both films, as they do not adversely affect the cell viability even at the higher concentration of Zn^2+^ ions [40]. This was probably due to the superhydrophobic properties of the DE@ZnS-ZnO@SA film, which partially reduced the interaction between the water and ZnS-ZnO granular film. Figure 3i,j show high-resolution SEM images of the two films immersed in a weak acid solution for 35 days. The SEM images show that the surface morphology of the two films did not change after 35 days of immersion. This was consistent with the morphology before immersion, indicating that the ZnS-ZnO granular film had a strong binding force with the substrate and excellent stability.

### 3.3. Chemical Durability and Corrosion Resistance of the DE@ZnS-ZnO@SA Film Surface

**Figure 4:**
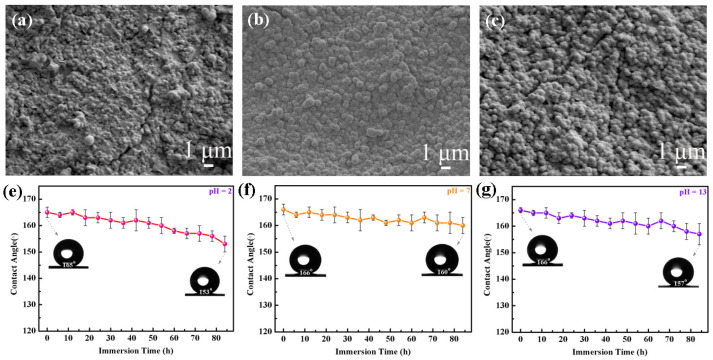

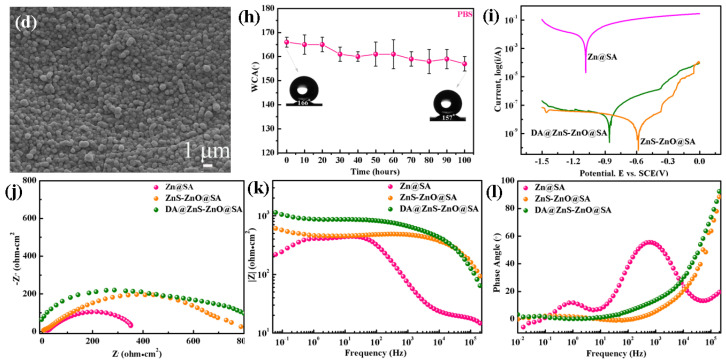
(**a**–**d**) The SEM images of the DE@ZnS-ZnO@SA film after the long-term corresponding soaking treatment. (**e**–**h**) The variation in the WCA with the immersion time of the DE@ZnS-ZnO@SA surface in acidic solution, 3.5 wt% NaCl aqueous solution, alkaline solution, and PBS solution (pH = 7.4). The down insets show the optical image of water droplets for the film surface before and after immersion of 84 h. (**i**) The measured potentiodynamic polarization curves of the Zn@SA, ZnS-ZnO@SA, and DE@ZnS-ZnO@SA films in a 3.5% NaCl solution. Nyquist and Bode diagrams of the samples. (**j**) Nyquist plots of the samples. (**k**) Bode plot of |Z| versus frequency. (**l**) Bode plots of the phase angle versus frequency.

It was necessary to test the chemical stability of the DE@ZnS-ZnO@SA film to prove that they could be used for implantable interventional devices. The DE@ZnS-ZnO@SA film was treated in different pH solutions to test the WCA (Figure 4e–g). The WCA test results showed that the WCA of the films decreased by 12° after soaking in an acidic environment (pH = 2) for 84 h, the WCA decreased by 6° after soaking in a neutral environment (pH = 7 sodium chloride solution) for 84 h, and the WCA decreased by 9° after soaking in an alkaline environment (pH = 13) for 84 h. Although the DE@ZnS-ZnO@SA film had poor stability in an acidic and alkaline environment, the WCA of the film surface was still greater than 150°. Our superhydrophobic composite coatings, when compared to the literature reports on single corrosion resistance, demonstrate enhanced multifunctional resistance to corrosion in extreme conditions such as strong acids, alkalis, and 3.5 wt% NaCl solutions [4,33]. The reason for this result was that the superhydrophobic film on the surface of the DE@ZnS-ZnO@SA film reduced the direct contact area between droplets with different pH values and ZnS-ZnO granular film, and blocked the reaction between the droplets and ZnS-ZnO granular film, thus ensuring that it had long-term chemical durability. This was confirmed by the Cassie model. According to the formula cosθ^C^ = f (cosθ^Y^ + 1) − 1, where f is the percentage of the apparent liquid–solid interface of the DE@ZnS-ZnO@SA surface (f ≤ 1), θ^Y^ is the WCA on the surface of the Zn substrate (WCA = 64°), and θ^C^ is the WCA on the surface of the DE@ZnS-ZnO@SA film (WCA = 166°). The solid–liquid contact area of the DE@ZnS-ZnO@SA film was only 2.0% by substituting these values into the formula of the Cassie model, and the maximum surface was covered by an air film, which greatly improved the corrosion resistance of the Zn substrate. Figure 4a–c show the SEM images of the DE@ZnS-ZnO@SA film immersed in different pH solutions for 84 h. The results show that the strong acid and strong alkali environments slightly corroded the granular film, resulting in the destruction of the SA and DE modified on the surface of the particles, but the neutral environment did not destroy the surface morphology of the film, keeping the surface wettability unchanged. Also, the film was immersed in phosphate buffer (pH = 7.4) to check the stability of the film in the physiological environment. With an increase in the immersion time, the WCA decreased only slightly (Figure 4h). Figure 4d is the SEM image of the film after soaking in PBS solution for 100 h. The image shows that the PBS immersion did not damage the surface morphology of the film, further proving that the superhydrophobic DE@ZnS-ZnO@SA film effectively protected the Zn substrate from corrosion. The corrosion or biodegradation rate of the implant met the rate of tissue healing. Therefore, the corrosion behavior of the material was further examined based on the immersion test. Figure 4i shows the polarization curves of the Zn@SA, ZnS-ZnO@SA, and DE@ZnS-ZnO@SA films. The Zn@SA film had a higher corrosion current (Icorr), indicating that the corrosion resistance of the Zn@SA film was weak. The corrosion current (Icorr) of the superhydrophobic ZnS-ZnO@SA and DE@ZnS-ZnO@SA films was small, indicating that the ZnS-ZnO@SA and DE@ZnS-ZnO@SA films exhibited optimum corrosion resistance in NaCl aqueous solution. Also, the corrosion behavior of each sample was further analyzed by electrochemical impedance spectroscopy (EIS). Figure 4j shows the Nyquist diagram of the EI obtained by each sample in a 3.5 wt% NaCl solution. The diameter of the impedance loops of the DE@ZnS-ZnO@SA film was much larger than that of the Zn@SA film, indicating that the superhydrophobic nano–submicron–micron particles significantly improved the corrosion resistance of the Zn matrix. Figure 4k,l present the impedance modulus and phase angle of the corresponding Bode modulus diagram. The impedance modulus and phase angle of the DE@ZnS-ZnO@SA film were greater than those of the Zn@SA film, indicating that the DE@ZnS-ZnO@SA film had the best electrochemical stability and corrosion resistance. This was because the low-surface-energy SA and the DE@ZnS-ZnO@SA microfilm with a multilayered nano–submicron–micron particle structure worked together to form a composite surface, capturing more air and forming a large air cushion in the void and groove structure, not only reducing the solid–liquid contact area but also preventing corrosive ions from penetrating into the coating [25,41]. Also, the multilayered nano–submicron–micron particle structure increased the diffusion path of the corrosive medium and finally achieved the long-term protection of the Zn substrate.

### 3.4. Wetting and Blood-Repelling Properties of the DE@ZnS-ZnO@SA Film Surface

**Figure 5:**
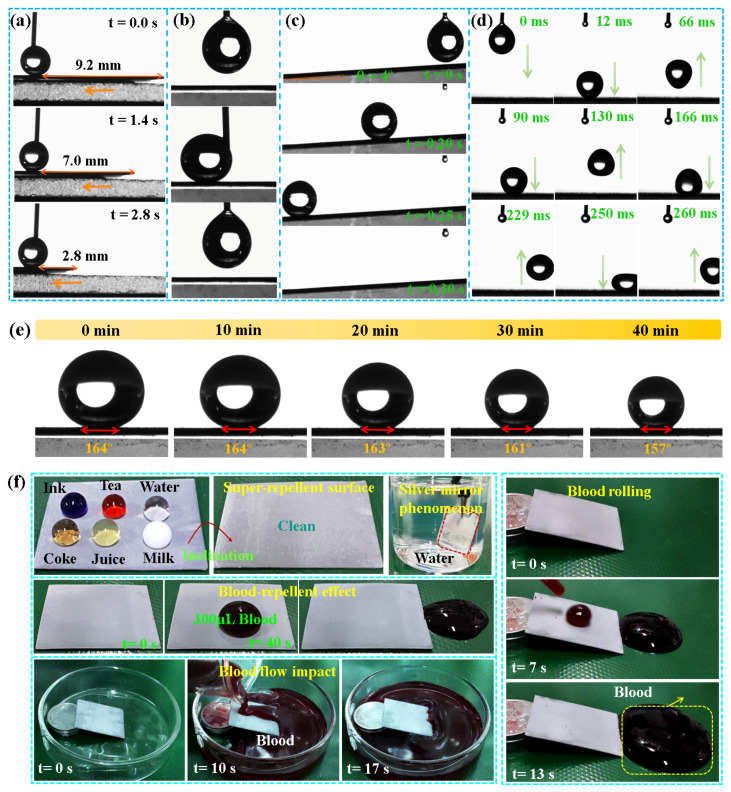
(**a**) A series of optical photos of the DE@ZnS-ZnO@SA film moving to the left under the squeeze of water droplets. (**b**) A series of optical photos of a 5 μL water droplet approaching, touching, and leaving. (**c**) A 5 μL water droplet falling and rolling behavior from a slightly tilted film surface. (**d**) A series of snapshots of a water droplet impacting the DE@ZnS-ZnO@SA film with arrows indicating the direction of its instantaneous movement. (**e**) Successive snapshots of the evaporation of 8 μL water droplets on the DE@ZnS-ZnO@SA film surface. (**f**) The wetting behavior of various droplets on the surface of the DE@ZnS-ZnO@SA film.

Adhesion is an important evaluation parameter for the application of superhydrophobic film [25,42]. Next, 5 μL of water droplets at the tip of the syringe were attached to the surface of the DE@ZnS-ZnO@SA film to maintain a constant position, and the position of the sample was moved to the left (Figure 5a). Even at a 6.4 mm distance, the water droplets were still in the original position, indicating that the surface of the DE@ZnS-ZnO@SA film had low adhesion and uniform superhydrophobic distribution. Figure 5b shows a series of optical images of 5 μL water droplets hanging on the syringe, approaching, touching, and leaving the superhydrophobic DE@ZnS-ZnO@SA surface. The syringe gradually moved down to the surface of the DE@ZnS-ZnO@SA sample. The water droplets remained spherical even when in close contact with the surface. After further extrusion, the water droplets rolled to the side of the sample, and no adhesion behavior was observed. As the syringe moved upwards, the water droplets completely left the array surface, and no residual liquid was still attached to the tip. These results indicated that the DE@ZnS-ZnO@SA surface had a low adhesion to water. Figure 5c shows the test results of the low adhesion between the DE@ZnS-ZnO@SA surface and the water droplets. The water droplets quickly left the DE@ZnS-ZnO@SA surface with an inclination angle of 4° without residual traces, indicating excellent superhydrophobicity. Also, a high-speed camera was used to record the bouncing process of the water droplets impacting the surface of the DE@ZnS-ZnO@SA sample. Figure 5d shows that, when the water droplets impacted the superhydrophobic surface, they rebounded on the surface seven times without becoming wet, until they finally rolled off of the surface due to the extremely low adhesion to water. The experiment also used the droplets’ evaporation behavior to prove the sample’s adhesion (Figure 5e). The droplets on the surface of the DE@ZnS-ZnO@SA sample always maintained a ‘standing posture’ throughout the test. During the 40 min test period, although the droplets gradually evaporated, the WCA remained >157°, indicating that the surface had a highly stable superhydrophobicity. Meanwhile, this constant contact angle (CCA) evaporation mode indicated very low water adhesion. These results may be because the presence of ZnS-ZnO microspheres created a uniform and reasonable micro/nano-roughness, resulting in more gaps or cavities on the surface. This structure effectively captured a large amount of air into the film, thereby reducing the effective area of the solid–liquid contact and effectively preventing the infiltration of water droplets in the film gap, resulting in water droplets being suspended on the rough surface. On the other hand, the modification of the SA and DE reduced the film’s surface energy, making the film have a lower adhesion. Therefore, the DE@ZnS-ZnO@SA surface with low adhesion and good bounce performance was expected to effectively remove surface contaminants and achieve self-cleaning properties similar to lotus leaves. Meanwhile, lower adhesion also allowed blood-containing bacteria or viruses to bounce off of the superhydrophobic surface while hitting the surface completely, reducing the need for cleaning and disinfection.

The implantable interventional devices exposed to various environments are easily contaminated by solid or liquid pollutants. Thus, it is necessary to test the self-cleaning ability of the DE@ZnS-ZnO@SA film’s surface. When multiple droplets were dropped on the surface of the superhydrophobic DE@ZnS-ZnO@SA film, water droplets formed spherical droplets on the surface. After tilting the sample, the droplets easily rolled off from the surface of the film without leaving any traces. Also, when the DE@ZnS-ZnO@SA film was immersed in water, a mirror effect on the surface of the film was clearly observed (Figure 5f). This further proved the presence of the Cassie–Baxter system on the surface of the superhydrophobic film, which was due to the multilayer structure of the film surface that helped retain air in the gaps between the microspheres [41,43]. The trapped air repelled the liquid, making it difficult for the liquid to penetrate the film surface. Meanwhile, the potential application of the superhydrophobic DE@ZnS-ZnO@SA film in repelling blood and reducing blood clotting was also investigated. The blood easily slipped from the surface of the superhydrophobic film without leaving any residue. Even when a large amount of blood was dumped on the surface of the superhydrophobic DE@ZnS-ZnO@SA film, the film exhibited a strong repulsive ability. The DE@ZnS-ZnO@SA film still had an excellent blood-rejection ability (Figure 5f), even if the volume of blood was increased (400 μL). This preliminary result indeed shows the potential benefits of the blood-repellent ability of the superhydrophobic surface for implanting interventional devices. This will reduce the adsorption and activation of blood components (such as red blood cells and platelets) on the surface of the device, which will help reduce the risk of thrombosis. At the same time, it may also reduce the immune response and inflammation caused by the implant and improve its biocompatibility. This conjecture will be verified in subsequent sections.

### 3.5. Hydrogen Sulfide Detection and Histocytocompatibility

**Figure 6:**
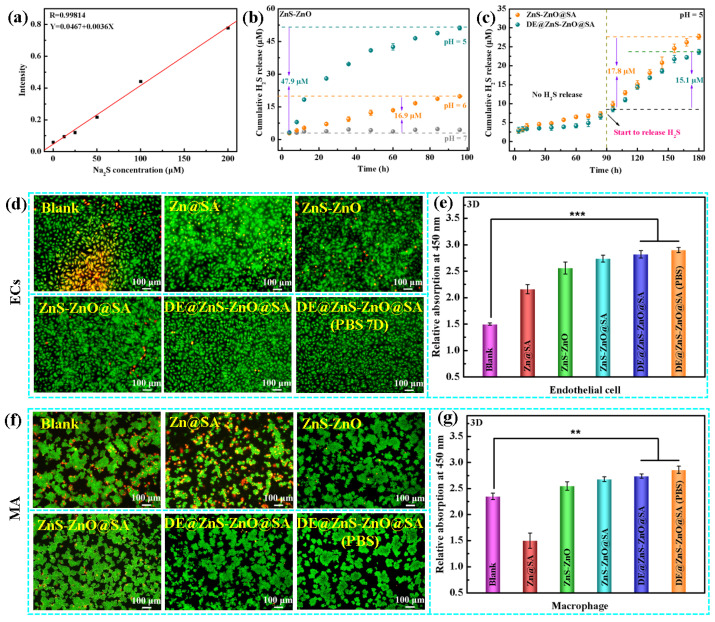
(**a**) The standard curve was generated using Na_2_S as an H_2_S donor. (**b**) The cumulative H_2_S gas release of the ZnS-ZnO film in solutions with different pH values (pH = 5, 6, and 7). (**c**) The cumulative H_2_S gas release of the ZnS-ZnO@SA and DE@ZnS-ZnO@SA film surfaces in solution at a pH = 5. (**d**) Fluorescent staining of the living (green) and dead (red) cells of the ECs. (**e**) CCK-8 activity assay of the ECs. (**f**) Fluorescent staining of the living (green) and dead (red) cells of the MA. (**g**) CCK-8 activity assay of the MA. All of the samples and blank groups were cultured in an acidic cell environment (pH = 6). The blank group was a normal cell group cultured in an acidic environment (pH = 6) without any sample. ** *p* < 0.01 and *** *p* < 0.001.

The DE@ZnS-ZnO@SA film had an H_2_S smell during the long-term immersion in strong acid, which was probably due to the reaction of the ZnS particles with hydrochloric acid. As a new type of gas signaling molecule, H_2_S plays an important protective role in the cardiovascular system. Thus, the release characteristics of H_2_S gas in vitro in samples with different structures and wettability in acidic environments were further studied. Figure 6a shows the standard curve of the H_2_S gas. First, the H_2_S release properties of the ZnS-ZnO film (160 °C) at different pH values (5, 6, and 7) was tested. The H_2_S concentration was almost undetectable at a pH = 7, and a large amount of H_2_S gas was produced under the acidic conditions of a pH = 6 and pH = 5, and the release rate and release amount of H_2_S increased with a decrease in the pH (Figure 6b). Compared with the H_2_S release time reported in the literature [44,45], the prepared ZnS-ZnO film could control the release of H_2_S for a long time, which greatly improved the efficacy of H_2_S as a therapeutic agent. These results showed that, in the presence of H^+^ ions, the ZnS particles reacted with H^+^ to produce H_2_S gas, and the higher the content of ZnS, the more H_2_S gas was released. Next, the ability of the superhydrophobic ZnS-ZnO@SA and DE@ZnS-ZnO@SA films to release H_2_S in an acidic environment (pH = 5) was tested. The superhydrophobic film began to release H_2_S gas after soaking in an acidic environment for 90 h. The cumulative release of H_2_S on the surface of the DE@ZnS-ZnO@SA film was slightly lower compared with that of the ZnS-ZnO@SA film (Figure 6c), which was probably caused by the presence of the hydrophobic drug DE. Furthermore, compared with the cumulative release of H_2_S on the superhydrophilic ZnS-ZnO film (160 °C), the superhydrophobic surface inhibited the release of H_2_S, and the cumulative release of H_2_S was 2.3 times lower than that of the superhydrophilic surface. This was probably due to the modification of the SA and DE with low surface energies, due to which the H^+^ did not directly react with the ZnS and reduced the release of the H_2_S gas. Also, due to the roughness of the film, the surface of the film modified by the SA and DE became a composite interface. The trapped air between the particles reduced the contact area between the film particles and the acidic aqueous solution, thereby reducing the release of H_2_S.

However, an acidic extracellular microenvironment in the lesion site affects the activity and function of the ECs and macrophages (MAs). The lesion environment also reduces the production of endogenous H_2_S, reducing its protection on the cells. Therefore, improving the cell microenvironment and restoring the activity and function of the ECs and MAs are essential to heal the lesion. H_2_S plays a vital regulatory role in cell function due to its anti-inflammatory, antioxidant, cytoprotective, and pro-angiogenic effects. Consequently, this study explored the effect of the films’ released H_2_S in an acidic environment (pH = 6) on the growth of the ECs and MAs. The acidic EC and MA cell suspensions were inoculated on the surfaces of the Zn@SA, ZnS-ZnO, ZnS-ZnO@SA, and DE@ZnS-ZnO@SA films, respectively. Figure 6d shows the fluorescence images of the ECs after 3 days of culture in an acidic environment (pH = 6). A large amount of apoptosis occurred in the ECs of the blank group (normal EC cell group cultured in an acidic environment (pH = 6) without any sample). Due to the release of Zn^2+^ in the acidic environment (pH = 6), the number of ECs undergoing apoptosis in the sample group (Zn@SA) was less than that in the blank group (normal EC cell group cultured in acidic environment (pH = 6) without any sample), retarding cell death. The ZnS-ZnO film sample group also promoted the proliferation of ECs compared with the blank group (normal EC cell group cultured in acidic environment (pH = 6) without any sample). The total apoptosis rate of the cells was also reduced. The sample group (DE@ZnS-ZnO@SA) soaked in PBS for 7 days had the best effect. The results confirmed that the combined action of Zn^2+^ and H_2_S can protect the vascular ECs from damage due to the acidic environment (pH = 6), restoring their proliferation and biological activity. This was consistent with the CCK-8 results (Figure 6e). In addition, similar conclusions can be drawn from the results of the MAs. Figure 6f,g show that, compared with the blank group (normal MA cell group cultured in acidic environment (pH = 6) without any sample) and the sample group, the appropriate administration of exogenous H_2_S effectively reduces the damage to the MAs, inhibits apoptosis, promotes their proliferation, and restores their biological characteristics.

### 3.6. Anticoagulation

**Figure 7:**
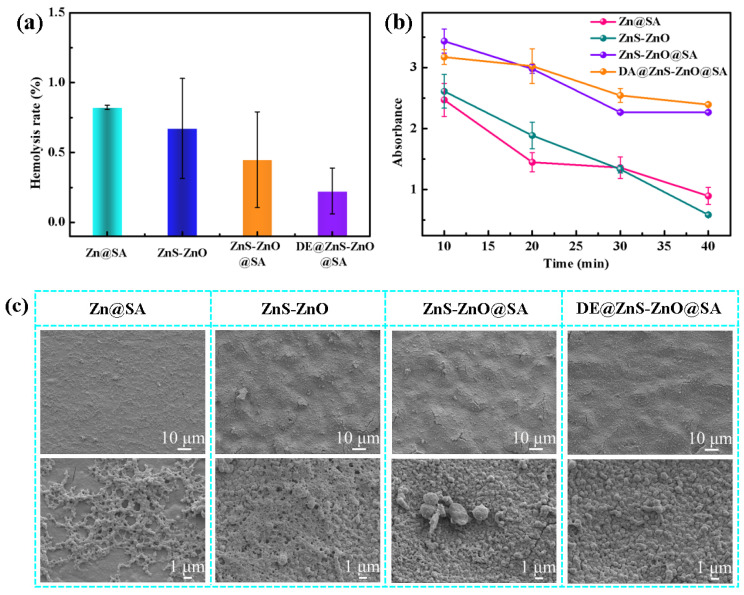

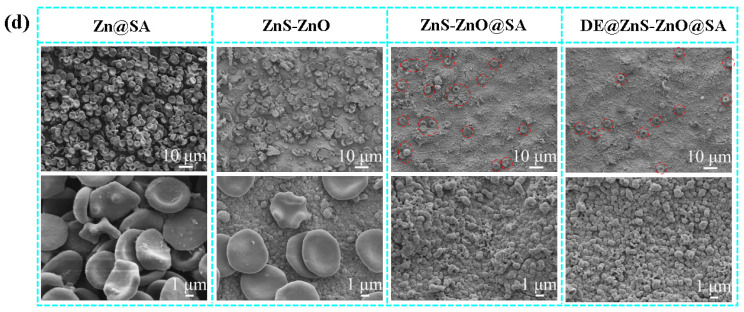
In vitro anticoagulation evaluation of the sample surfaces. (**a**) Hemolysis test on the surface of each sample. (**b**) The dynamic coagulation time of the surface of each sample. (**c**) SEM images of platelet adhesion and activation. (**d**) SEM image of erythrocyte adhesion and activation. In the red circle are red blood cells.

During the service of implantable interventional materials, the particles in the film can enter the blood tissue, causing hemolysis and cytotoxicity. Therefore, the hemolysis test and cytotoxicity test were performed to verify the biocompatibility of the film. The hemolysis rate of all of the samples was <1%, which was much lower than the international standard (less than 5%), indicating that the DE@ZnS-ZnO@SA film exhibited no hemolysis toxicity, had little effect on the blood cells, and possessed good blood compatibility (Figure 7a). The construction of surfaces with long-term anticoagulant properties is a critical factor in preventing implant failure [46,47]. Therefore, a dynamic coagulation experiment was performed. The absorbance data were used to represent the relative amount of uncoagulated blood on the material surface after blood contact. The smaller the absorbance value, the higher the relative coagulation volume and coagulation degree. Figure 7b shows that the absorbance values of the superhydrophobic ZnS-ZnO@SA and DE@ZnS-ZnO@SA films were higher than those of the Zn@SA and ZnS-ZnO films, indicating that the ZnS-ZnO film modified by the SA and DE improved the anticoagulant performance of the Zn substrate, prolonged the dynamic clotting time, and improved the blood compatibility of the Zn substrate. The DE@ZnS-ZnO@SA film had the highest absorbance, indicating the excellent anticoagulant properties. The combined effect of the low surface energy of the SA and DE reduced the adsorption capacity of the film to the blood component and improved the anticoagulant properties of the ZnS-ZnO film. The samples were incubated with 400 μL of platelet-rich plasma and fresh whole blood for 40 min to study the anticoagulant properties of the samples. Figure 7c shows that a large number of platelets adhered to the surface of the Zn@SA and ZnS-ZnO films, and were severely activated, showing a spreading and fully spreading state. On the contrary, the number of platelets adhering to the surface of the superhydrophobic ZnS-ZnO@SA film was significantly reduced, and the platelet morphology remained round without obvious deformation or pseudopods. The surface of the DE@ZnS-ZnO@SA film did not have any adhered platelets, and the original morphological characteristics of the film were still maintained, indicating that the superhydrophobic ZnS-ZnO@SA film further improved the anticoagulant performance of the film after drug loading. Figure 7d shows the SEM results of the sample surface after co-culturing with fresh whole blood for 40 min. The red blood cells and fibrinogen adhered to the surface of the Zn@SA and ZnS-ZnO films, and the red blood cells were deformed, indicating that the surfaces of the Zn@SA and ZnS-ZnO films were prone to coagulation. The ZnS-ZnO@SA film had a certain anti-adhesion due to the introduction of low-surface-energy SA; thus, the surface adhesion of the red blood cells was significantly reduced, and the coagulation was weakened. However, a small amount of red blood cells appeared on the surface of the DE@ZnS-ZnO@SA film, and the red blood cells did not deform, indicating that the DE@ZnS-ZnO@SA film had good anticoagulant and antibiofouling properties. This was mainly due to the release of DE on the surface of the DE@ZnS-ZnO@SA film, inhibiting the adhesion and deformation of red blood cells and fibrinogen. This was consistent with the results of the platelet adhesion and activation experiments. Next, the platelet adhesion and whole-blood static culture experiments were performed on the samples soaked in PBS (pH = 7.4) for 7 days to further verify the long-term anticoagulant function of the film. The number of platelets adhered to the surface of the Zn@SA and ZnS-ZnO films significantly increased and was severely activated, being completely tilted on the surfaces of the films. The superhydrophobic ZnS-ZnO@SA film still reduced the number of platelet adhesions after 7 days of immersion; however, the platelet activation occurred at the position of the particle protrusion rather than in the air-protected depression area. For the superhydrophobic DE@ZnS-ZnO@SA film, only a few platelets adhered to the surface, and the platelets remained round in shape (Figure S3a). For the banded substance on the surface, this indicated the agglomeration of SA membranes after the PBS immersion, not the adhesion of platelets, which activated on the surface of the membrane. This was supported by the whole-blood experiments. The morphology of the red blood cells on the surfaces of the Zn@SA and ZnS-ZnO films after the PBS immersion changed significantly, and spinous red blood cells appeared. The superhydrophobic ZnS-ZnO@SA film surface reduced the number of adhered red blood cells; however, fibrinogen easily adhered to the particle surface (Figure S3b). In contrast, the DE@ZnS-ZnO@SA film had fewer red blood cells, and fibrinogen adhered to the surface, which further proved that the ZnS-ZnO@SA film further improved the anticoagulant performance of the film after drug loading. This was attributed to the modification of the superhydrophobic surface by a low-surface-energy material to form a composite surface. The air captured on the surface limited the surface area of the water in contact with the film, thereby effectively reducing the binding of the film to the protein.

**Figure 8:**
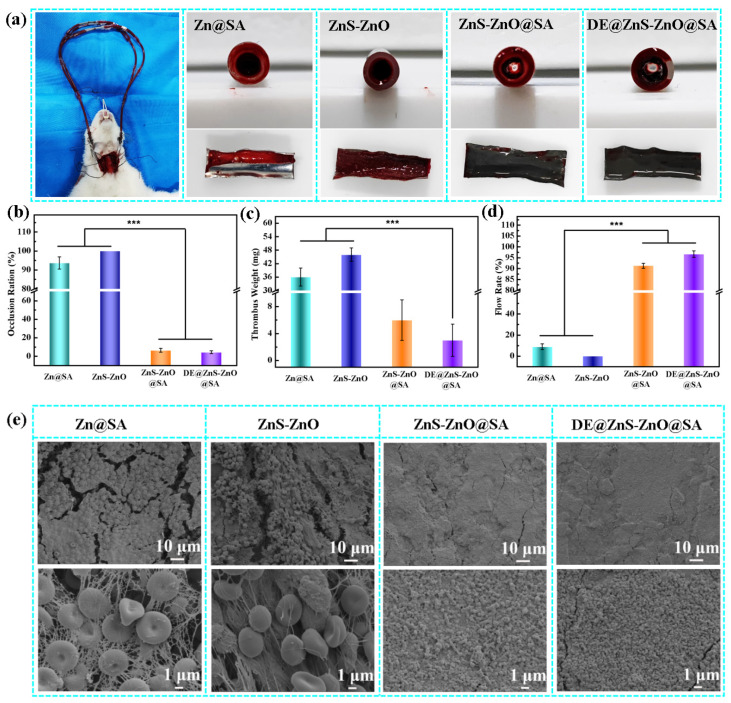
(**a**) Illustration of ex vivo dynamic blood circulation of New Zealand rabbit, photographs of the cross-sections of the samples in the catheters after 60 min of circulation, and the inner wall of the samples. (**b**) Relative flow rate of simulated body fluid with samples in the catheter at the end of the circulation. (**c**) Quantitative analysis of the thrombus weight on the surfaces. (**d**) Percentage of tubing occlusions determined by calculating the loss of the cross-section diameter. (**e**) SEM image of thrombi. *** *p* < 0.001.

Additionally, the antithrombotic ability of the samples was evaluated by using New Zealand female white rabbits as research objects. The samples (25 mm × 8 mm) were subjected to the whole-blood dynamic circulation experiment for 60 min (Figure 8a). The agglomerate formation on the inner wall of the film was evaluated from both qualitative and quantitative perspectives. The cross-section and inner wall surface photos of each sample show that different degrees of lumen loss occurred in the Zn@SA and ZnS-ZnO films, and prominent occlusion was observed in the cross-section. This is due to the sample surface’s poor anticoagulant performance, causing many platelets and red blood cells to aggregate to form a thrombus. However, no obvious thrombi were observed on the surfaces of the superhydrophobic ZnS-ZnO@SA and DE@ZnS-ZnO@SA films after 60 min of circulation, indicating that the superhydrophobic surfaces showed promising anticoagulant activity in the actual arteriovenous shunt model. This result was consistent with the calculations of each sample’s lumen loss rate, thrombus mass, and flow rate (See Figure 8b–d). The lumen loss rate of the superhydrophobic ZnS-ZnO@SA and DE@ZnS-ZnO@SA films was <6%, the thrombus mass was <6 mg, and the flow rate was >91%, which was significantly higher than that of the Zn@SA and ZnS-ZnO films, with a lumen loss rate of ≥93%, thrombus mass of ≥36 mg, and flow rate of ≤9%. The DE@ZnS-ZnO@SA film was revealed to have the best coagulation performance after loading the anticoagulants. Further observation by scanning electron microscopy showed that many red blood cells, platelets, and fibrin networks were attached to the surface of the Zn@SA and ZnS-ZnO films, forming a serious thrombus. In contrast, significantly fewer platelets and red blood cells adhered to the surfaces of the superhydrophobic ZnS-ZnO@SA and DE@ZnS-ZnO@SA films, and no thrombi were formed (Figure 8e). The results show that the superhydrophobic film has a significant inhibitory effect on thrombosis. In addition, the half-in vivo whole-blood dynamic circulation experiment of the sample immersed in PBS (pH = 7.4) for 7 days was tested to verify if the sample displayed any anticoagulant effect after long-term immersion in a physiological solution. Figure S4a shows that the Zn@SA and ZnS-ZnO films were severely blocked after 1 h of circulation, and many adhered blood clots were formed on the inner wall. Although a small number of thrombi were detected on the surface of the ZnS-ZnO@SA film, there were no blood clots in most areas, indicating that the films also had excellent antithrombosis properties. In contrast, no obvious thrombosis was observed on the surface of the DE@ZnS-ZnO@SA film, and only a small amount of blood adhered to some places. This is because the long-term physiological solution immersion makes some film positions lose its superhydrophobicity, reducing the ability to resist blood adhesion. In addition, an analysis of the lumen loss rate, thrombus mass, and flow rate of each sample after immersion revealed that the immersed Zn@SA and ZnS-ZnO films completely lost their antithrombotic ability after 60 min of circulation compared with those before immersion. The antithrombotic ability of the superhydrophobic ZnS-ZnO@SA and DE@ZnS-ZnO@SA films were only slightly weakened compared to the previous ones, and could still inhibit blood clot formation (Figure S4b–d). Further, the antithrombotic performance of the superhydrophobic DE@ZnS-ZnO@SA film > ZnS-ZnO@SA film. This result was further supported by the SEM results of the sample surface (See Figure S4e). These qualitative and quantitative analysis results confirm that the superhydrophobic DE@ZnS-ZnO@SA film inhibits the formation of blood clots, blocks the activity of blood components involved in the formation of blood clots, and can be used for a long time.

### 3.7. Antibacterial

**Figure 9:**
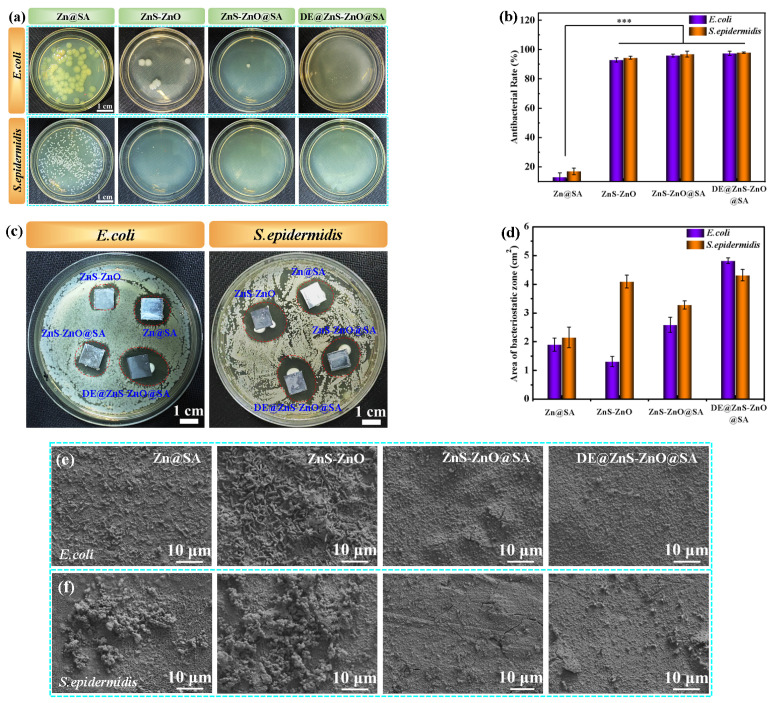
(**a**) Agar plate photographs and count number of viable *E. coli* and *S. epidermidis* colonies with different samples. (**b**) Inhibition rates of the coating against *E. coli* and *S. epidermidis.* (**c**) Inhibition zone of different samples against *E. coli* and *S. epidermidis*. (**d**) Measurement of bacteriostatic area with different samples. (**e**) SEM images of *E. coli* on the surface of each sample. (**f**) SEM images of *S. epidermidis* on the surface of each sample. *** *p* < 0.001.

A common problem in clinical practice is infection after the implantation of interventional devices. Thus, indwelling medical devices must have antibacterial properties to reduce bacterial infection. The antibacterial properties of the samples were evaluated by the bacteriostatic ring method. Figure 9a shows that *E. coli* and *S. epidermidis* are densely distributed around the Zn@SA film, indicating that the antibacterial effect of the Zn@SA film was doubtful. In contrast, the superhydrophilic ZnS-ZnO film, superhydrophobic ZnS-ZnO@SA film, and DE@ZnS-ZnO@SA film have potent antibacterial zones with an inhibition diameter > 2.1 cm. This result indicated that the ZnS-ZnO film could kill the bacteria inoculated under the sample and all of the colonies around it by releasing Zn^2+^ ions into the culture medium. Additionally, the superhydrophobic DE@ZnS-ZnO@SA film was found to have the best inhibitory effect on bacteria by further comparing the antibacterial ability of composite films with a different wettability. The superhydrophobic surface reduced bacterial adhesion, thereby inhibiting bacterial growth. Similarly, this result was further supported by the surface inhibition area of the sample (Figure 9b). The antibacterial properties of the samples against *E. coli* and *S. epidermidis* were quantitatively studied by the standard colony counting method. Figure 9c,d show many colonies on the surface of the Zn@SA film. Thus, the antibacterial rate of *E. coli* and *S. epidermidis* was effectively 0%. The ZnS-ZnO film surface displayed a 93% antibacterial effect on *E. coli* and *S. epidermidis.* This is mainly because the high Zn^2+^ concentration led to a difference in the ion concentration inside and outside of the bacterial cell membrane, destroying the bacterial nutrients and causing their death. In contrast, the number of bacterial colonies on the surfaces of the superhydrophobic ZnS-ZnO@SA and DE@ZnS-ZnO@SA films were significantly reduced, and the antibacterial rate against *E. coli* and *S. epidermidis* was about 96%. This is because the SA modified on the ZnS-ZnO film surface also has an antibacterial effect [48]. Thus, the combined action of the Zn^2+^ and SA imparts the superhydrophobic film with a better antibacterial ability. In addition, in order to further verify the effect of the sample on the adhesion of *E. coli* and *S. epidermidis*, the adhesion of the two bacteria on the surface of each sample was observed by SEM. The experimental results are shown in Figure 9e,f. It can be seen from the figure that the surfaces of the Zn@SA and ZnS-ZnO films were adhered by a large number of bacteria, but no bacterial adhesion was observed on the surfaces of the ZnS-ZnO@SA and DE@ZnS-ZnO@SA films, indicating that superhydrophobicity has the ability to resist bacterial adhesion.

### 3.8. Mechanism Analysis

**Figure 10:**
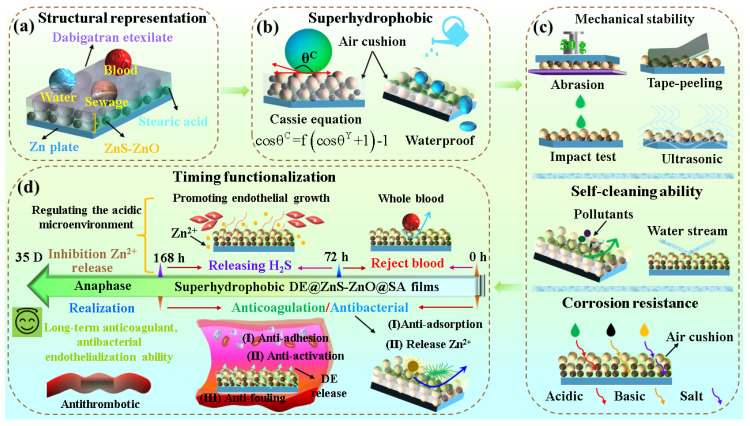
(**a**–**d**) Mechanistic description of the superhydrophobic, waterproof, mechanical stability, self-cleaning, corrosion resistance, blood-repellent, anticoagulation, antibacterial, and endothelialization-promoting properties of the sequential functionalization of the superhydrophobic DE@ZnS-ZnO@SA film surface.

In this study, a robust superhydrophobic DE@ZnS-ZnO@SA film was prepared by a simple strategy (Figure 10a). The film has extremely low adhesion, excellent mechanical stability, corrosion resistance, is multi-liquid-repellent, and has long-term antithrombotic and antibacterial properties. Among these, the main reason for the low adhesion and water repellency of the film is the presence of multilayer nano–submicron–microspheres, which improves the film’s surface roughness. After co-modification with low-surface-energy SA and DE, a substantial amount of air is captured in the “gap” of the nano–submicron–micron particles, forming a solid air–solid composite interface and reducing the solid–liquid contact area and adhesion (Cassie model). Further, the Cassie equation indicates that the solid–liquid contact area of the water droplets in the DE@ZnS-ZnO@SA film only accounts for 2.0% of the total contact area; the air part accounts for a large proportion of the contact surface. Therefore, the material’s surface has a strong water repellency, which leads to the small frictional resistance of the fluid flowing through the surface. In addition, the modification by the SA and DE also reduced the adhesion of the film and further increased the water repellency (Figure 10b). For the reasons discussed above and the in situ growth of multilayer microspheres on the surface of the Zn substrate, the film still has excellent mechanical stability, durability, and self-cleaning properties under various physical stimuli (Figure 10c). Further, it can repel various solid and liquid pollutants. Moreover, the unique multilayer structure of the film, with dense nanospheres at the bottom, submicrospheres uniformly distributed in the middle, and microspheres dispersed at the top also prevents corrosive ions from penetrating through the film to the surface of the substrate [49]. This is because the multilayer structure increases the diffusion path of the corrosive medium, while the dense nanolayer prevents direct contact between the corrosive ions and the substrate, protecting the substrate in the long term. Moreover, the presence of low-surface-energy SA and DE prevents the shedding and dissolution of nano–submicron–micron particles, which again reduces the exposure of active sites on the ZnS-ZnO film surface. This, in turn, weakens the interaction between corrosive ions and the film and imparts it with excellent corrosion resistance, making it suitable for applications in blood-contact biomedical equipment. At the initial stage of implanting the blood-contact device into the blood vessel (before 90 h), the low adhesion of the superhydrophobic surface repels the blood, preventing any damage that could be caused by the interaction of blood cells on the film surface, achieving good blood compatibility. Meanwhile, the shear force and viscous resistance of the blood flow through the superhydrophobic surface are significantly reduced due to its reduction in water resistance (Figure 10d) [50].

The superhydrophobic surface exhibits good blood repellency, ensuring no visual loss of blood transport. Blood droplets sliding out from the superhydrophobic surface can be easily removed without any residue by gentle cleaning. In addition, a low surface-free energy will reduce the adhesion of blood cells, platelets, and fibrinogen. In contrast, the air cushion formed by the multilayer nano–submicron–micron particle structure reduces the contact area between the blood cells and the surface. This mechanism limits the adhesion of the cell molecules and proteins to the surface and reduces the possibility of thrombotic events in the instrument [19,51]. Further, DE, a direct thrombin inhibitor, can directly inhibit thrombin activity in the body, block the conversion of fibrinogen to fibrin, and prevent the formation of thrombi or embolic substances in the body. It also inhibits free thrombin, fibrin-bound thrombin, and thrombin-induced platelet aggregation, displaying an excellent anticoagulant effect [52,53,54]. The improved DE@ZnS-ZnO@SA surface can not only effectively exclude blood, reduce the adsorption and activation of blood components, as well as the adsorption and aggregation of platelets, but also reduce its inflammatory response to surrounding tissues, thereby improving the biocompatibility of the implant. This design significantly improves the anticoagulant performance of the implant, which is of great significance for prolonging the service life of the implant and reducing postoperative complications [19,55].

Thus, the results discussed in this section indicate that the combination of the superhydrophobic and anticoagulant DE can achieve anticoagulation and antithrombotic effects for more than 168 h. Additionally, the rough structure and low adhesion of the DE@ZnS-ZnO@SA film surface provide a limited contact area between the microorganisms and superhydrophobic surfaces, reducing the fixation point of the bacterial cells. Further, the Zn^2+^ ions released from the coating kill the bacteria adhered to the film’s surface, imparting the film with excellent antibacterial properties. More importantly, the film spontaneously releases H_2_S gas and consumes H^+^ in the acidic environment (pH = 6) of the lesion, reducing the acidic microenvironment’s pH and promoting endothelial cell proliferation. Even after a long-term acid environment immersion for 35 days (pH = 6), the film also can inhibit the substantial release of Zn^2+^ ions. In summary, the DE@ZnS-ZnO@SA film first utilizes the blood-repellent behavior of the superhydrophobic surface and the release of the anticoagulant DE to achieve a long-term anticoagulant function. Then, the in situ release of H_2_S gas was achieved by using the acidic microenvironment, which provided a continuous endothelialization effect. Finally, under the synergistic effect of low-surface-energy SA and a multilayer nano–submicron–micron particle structure, the uncontrollable release of Zn^2+^ can be inhibited and its toxicity can be avoided. This time-sequential functionalization surface will become a typical long-term use of blood-contact stents in the future.

## 4. Conclusions

This study reports the preparation of a superhydrophobic DE@ZnS-ZnO@SA film on the surface of the Zn substrate by a simple and green one-step hydrothermal method and dip-coating technology. The resulting non-toxic film exhibited good biocompatibility, high mechanical strength, corrosion resistance, and self-cleaning properties. This is mainly due to a multilayer dense microsphere structure and low-surface-energy SA, which makes the film’s surface present a Cassie state, forming a stable gas–solid interface on the surface and reducing the adhesion of the film. The combination of low adhesion and anticoagulant DE makes the film exhibit sequential functionality in an acidic environment (pH = 6). First, the long-term anticoagulant performance of the film was achieved by the combined action of the blood-repellent and the anticoagulant properties of the superhydrophobic surface and DE, respectively. Meanwhile, the superhydrophobic surface’s low adhesion and Zn^2+^ release also exhibited excellent inhibitory and antibacterial activity. Second, ZnS particles on the film’s surface react with H^+^ in an acidic environment (pH = 6) to generate H_2_S gas, promoting the proliferation of ECs. Finally, the synergistic effect of the superhydrophobic surface as a corrosion inhibitor and the layered, dense micro/nanosphere structure inhibits the uncontrollable release of Zn^2+^ ions on the film’s surface in the long-term blood immersion. In summary, this timing of the multifunctional DE@ZnS-ZnO@SA film effectively improves the long-term anticoagulant, antibacterial, and pro-endothelialization functions of blood-contact devices and provides a new functional integration idea for the surface modification of blood-contact devices.

## Data Availability

Data for this work are available from the corresponding author on reasonable request.

## Supplementary Materials

The following supporting information can be downloaded at: https://www.mdpi.com/article/10.3390/biom14081041/s1. The SEM images and WCA images of the prepared ZnS-ZnO film under the hydrothermal temperature of 160 °C for 4, 8, 12, and 16 h, respectively; the SEM images, high-resolution XPS spectra, EDS, and surface element distribution mapping of the ZnS−ZnO film before and after treatment; the SEM images of erythrocyte and platelet adhesion and the activation of each sample immersed in PBS for 7 days; the whole-blood dynamic circulation experiment of the samples soaked in PBS for 7 days.
