# Supplementary material for: Preparation of Time-Sequential Functionalized ZnS-ZnO Film for Modulation of Interfacial Behavior of Metals in Biological Service Environments
Source: Biomolecules. 2024 Aug 22;14(8):1041. doi: 10.3390/biom14081041 (PMC11352253; doi:10.3390/biom14081041)
Supplement: Supplementary file 1 [file biomolecules-14-01041-s001.zip › biomolecules-3153768-supplementary.pdf]

Supporting Information

Preparation of Time-Sequential Functionalized ZnS-ZnO Film for Modulation of Interfacial Behaviour of Metals in Biological Service Environments.

Jianwen Zhang[1], YuJie Tang[1], XiaoWa Gao[1], Xinyu Pei[1], Yajun Weng[2], Junying Chen[1*]

1. Key Laboratory of Advanced Technology of Materials, Ministry of Education, School of Materials Science and Engineering, Southwest Jiaotong University, Chengdu 610031, PR China.

2. Institute of Biomedical Engineering, College of Medicine, Southwest Jiaotong University, Chengdu 610031, PR China.

*** Corresponding author**

Email address: chenjunying@swjtu.edu.cn (J. Y. Chen)

[*]Corresponding author.
E-mail address: chenjunying@swjtu.edu.cn (J. Y. Chen)
E-Tel: +86 28-87601149; Fax: +86 28-87601149

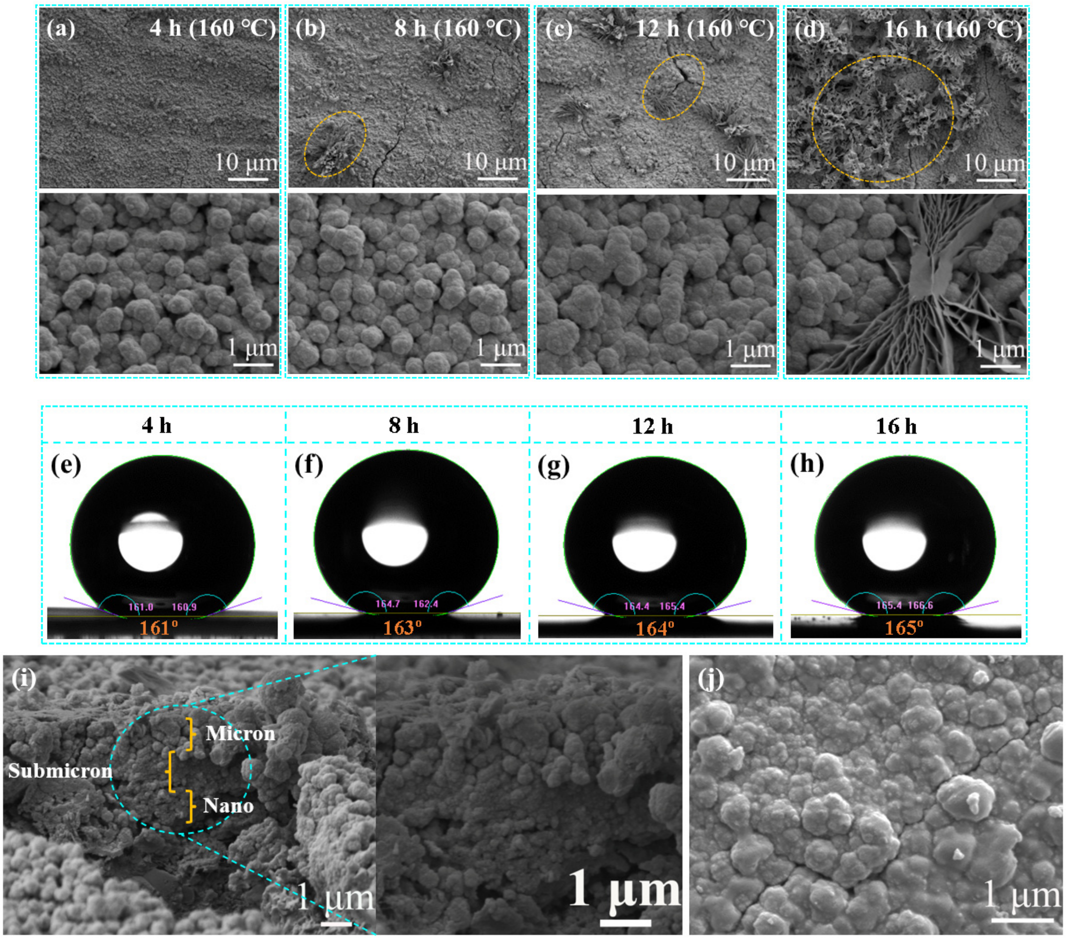

Figure S1. (a-d) SEM images of the prepared ZnS-ZnO film under hydrothermal temperature of 160 °C for 4, 8, 12, and 16 h, respectively. (e-h) Optical photograph of water droplet of ZnS-ZnO films modified with SA under hydrothermal temperature of 160 °C for 4, 8, 12, and 16 h, respectively. (i) The SEM image of the cross section of ZnS-ZnO (160 °C) film. (j) The SEM image of ZnS-ZnO (160 °C) film modified by dabigatran ester (DE). (The concentration of DE was 800 μg/mL)

Table S1

Sample	Zn	100 °C	120 °C	140 °C	160 °C	180 °C
WCA (°)	64	4	7	6	8	6

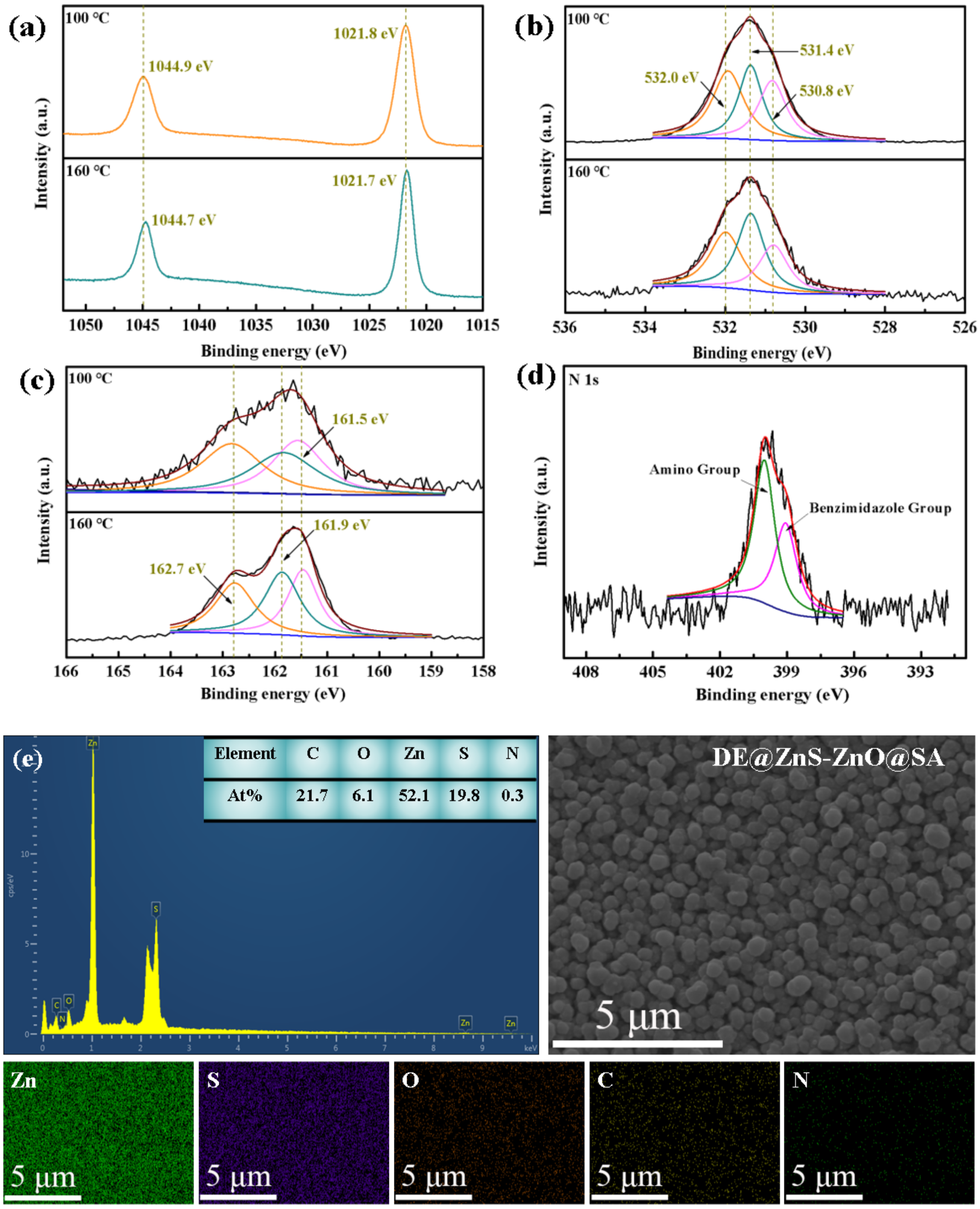

Figure S2. The corresponding high-resolution spectra of (a) Zn 2p, (b) O 1s, (c) S 2p and N 1s speaks for ZnS-ZnO film with hydrothermal temperatures of 100 °C and 160 °C. (e) The SEM image, Energy dispersive spectrum (EDS) and surface element distribution mappings of the superhydrophobic DE@ZnS-ZnO@SA films surface.

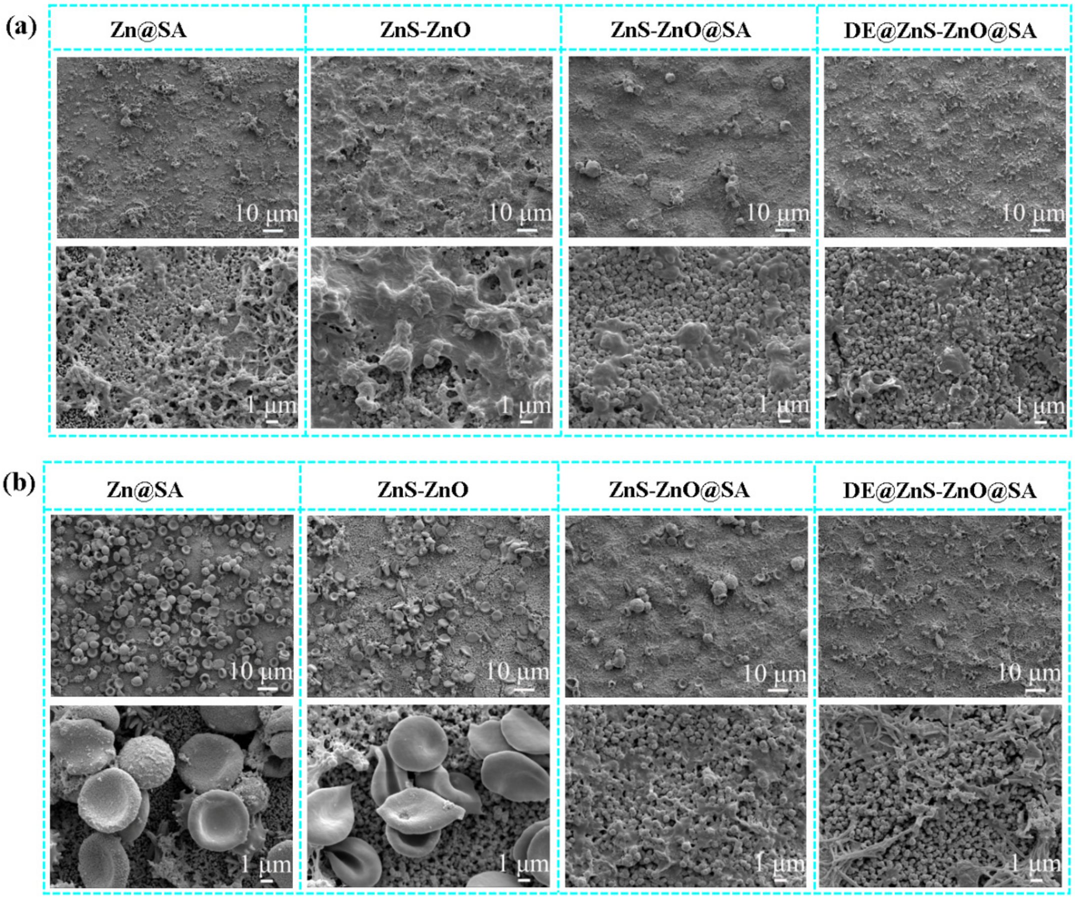

Figure S3. (a) SEM images of platelet adhesion and activation of each sample immersed in PBS for 7 days. (b) SEM image of erythrocyte adhesion and activation of each sample immersed in PBS for 7 days.

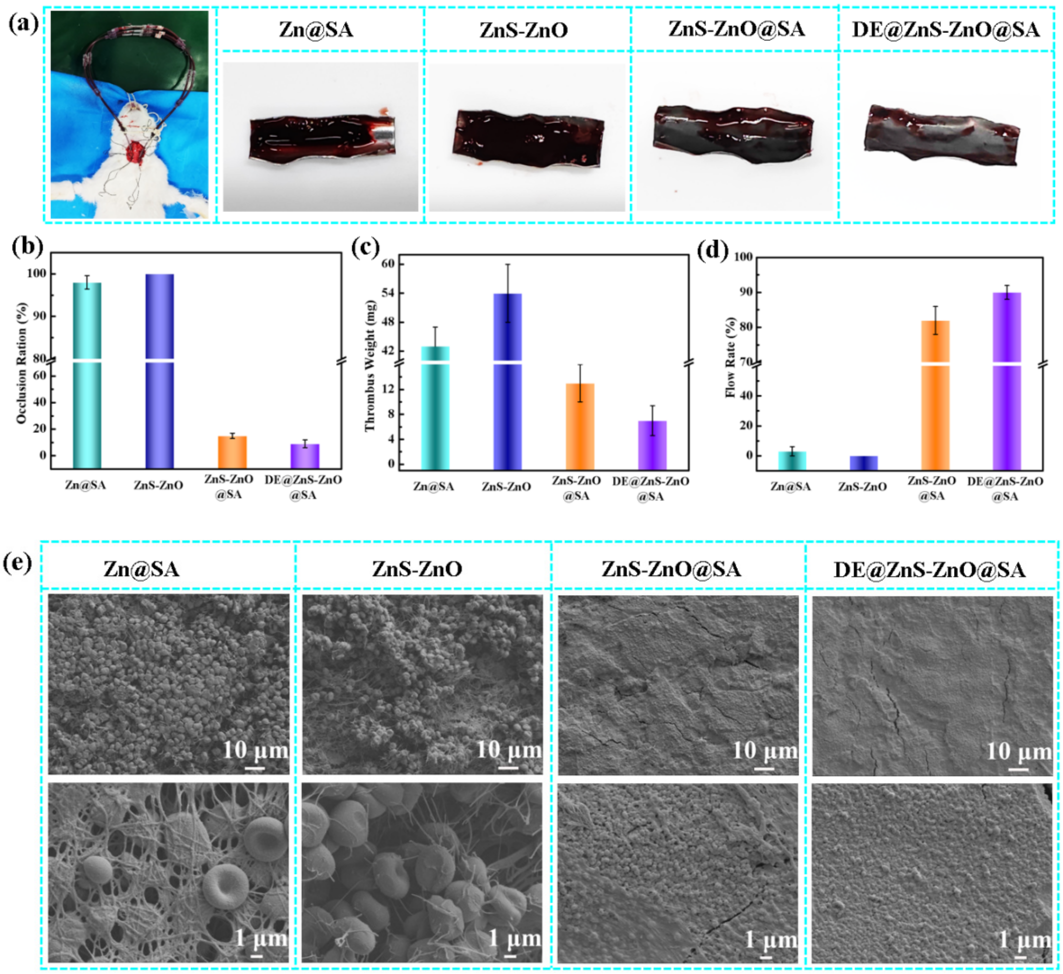

Figure S4. (a) The ex vivo circulation of the heparinized extracorporeal circulation catheter with the samples of Zn@SA, ZnS-ZnO, ZnS-ZnO@SA and DE@ZnS-ZnO@SA were immersed in PBS for 7 days, and photographs of the samples in the catheters after 60 min circulation and the inner wall of the samples. (b) Percentage of tubing occlusions determined by calculating the loss cross section diameter. (c) Quantitative analysis of the thrombus weight on the surfaces. (d) Relative flow rate of simulated body fluid with samples in the catheter at the end of the circulation. (e) SEM image of thrombi.
